# The Tandem Repeats Enabling Reversible Switching between the Two Phases of β-Lactamase Substrate Spectrum

**DOI:** 10.1371/journal.pgen.1004640

**Published:** 2014-09-18

**Authors:** Hyojeong Yi, Han Song, Junghyun Hwang, Karan Kim, William C. Nierman, Heenam Stanley Kim

**Affiliations:** 1 Department of Biosystems and Biotechnology, Korea University, Seoul, Korea; 2 Department of Biomedical Sciences, Korea University, Seoul, Korea; 3 J. Craig Venter Institute, Rockville, Maryland, United States of America; Uppsala University, Sweden

## Abstract

Expansion or shrinkage of existing tandem repeats (TRs) associated with various biological processes has been actively studied in both prokaryotic and eukaryotic genomes, while their origin and biological implications remain mostly unknown. Here we describe various duplications (*de novo* TRs) that occurred in the coding region of a β-lactamase gene, where a conserved structure called the omega loop is encoded. These duplications that occurred under selection using ceftazidime conferred substrate spectrum extension to include the antibiotic. Under selective pressure with one of the original substrates (amoxicillin), a high level of reversion occurred in the mutant β-lactamase genes completing a cycle back to the original substrate spectrum. The *de novo* TRs coupled with reversion makes a genetic toggling mechanism enabling reversible switching between the two phases of the substrate spectrum of β-lactamases. This toggle exemplifies the effective adaptation of *de novo* TRs for enhanced bacterial survival. We found pairs of direct repeats that mediated the DNA duplication (TR formation). In addition, we found different duos of sequences that mediated the DNA duplication. These novel elements—that we named SCSs (same-strand complementary sequences)—were also found associated with β-lactamase TR mutations from clinical isolates. Both direct repeats and SCSs had a high correlation with TRs in diverse bacterial genomes throughout the major phylogenetic lineages, suggesting that they comprise a fundamental mechanism shaping the bacterial evolution.

## Author Summary

β-lactamases can adapt to new antibiotics by mutations in their genes. The original and the extended substrate spectrums of β-lactamases define two phases of catalytic activity, and the conversion by point mutations is unidirectional from the initial to the new spectrum. We describe duplication mutations that enable reversible switching between the substrate spectrums, increasing the adaptability of the bacterium. We provide evidence supporting that two distinct groups of short sequences mediated the formation of DNA duplications in β-lactamases: direct repeats and novel elements that we named, SCSs (same-strand complementary sequences). Our study suggests that DNA duplication processes mediated by both direct repeats and SCSs are not just limited to the β-lactamase genes but comprise a fundamental mechanism in bacterial genome evolution.

## Introduction

As a ubiquitous feature of genomes, tandem repeats (TRs) are the sites at which recombination or replication slippage can occur [1]–[3]. Changes in the number of repeat units can confer phenotypic variability in eukaryotes, such as plasticity in skeletal morphology and tuning of the circadian rhythm, and are critical in repeat expansion diseases in humans, such as Huntington's disease [1]. In microorganisms, changes in TRs are the basis for one of the simplest and most prevalent reversible stochastic switching mechanisms, which is commonly known as “phase- or antigenic variation” [2]–[4]. Phase variation generally involves reversible switching that results in an “all-or-none” expressing phase of proteins, whereas antigenic variation alters the surface architecture of proteins that interact with the environment [3], [4].

Whereas biological consequences affected by alterations in preexisting TRs have been widely reported, processes underlying *de novo* TR formation and their biological implications have not been actively investigated. Along this line, it is intriguing that there have been reports of duplication mutations that occurred in the coding region of β-lactamase genes, expanding the substrate spectrum of the enzyme to include ceftazidime, a third-generation cephalosporin. A duplication of five amino acids was found in SHV-16 in a clinical isolate of *Klebsiella pneumoniae* [5] within the omega loop, which is a highly conserved structural domain constituting part of the active-site pocket [6]. In another report, a duplication of three residues was found in the omega loop of a class C β-lactamase in clinical strain *Enterobacter cloacae* GC1 [7]. The adaptation of β-lactamases in response to exposure to new antibiotics has been a major public health concern, and in almost all cases, point mutations resulting in an amino acid substitution in the enzymes have been responsible for this problem [8]–[10]. Although the biological legitimacy was unclear, the two cases of rare duplication mutations suggested that β-lactamases have potential as an excellent subject to investigate the nature of *de novo* TRs in connection with the evolution of the drug resistance.

In this study, we describe eleven *de novo* TRs that can occur in the coding region of a β-lactamase gene and allow genetic toggling when coupled with reversion, adjusting the substrate spectrum of the β-lactamase to a different β-lactam antibiotic challenge. At the DNA level, we describe pairs of direct repeats and a novel group of duo elements that we found instrumental in DNA duplication. We then note our findings of a high correlation that exists between direct repeats and TRs and also between the novel duo elements and TRs, supporting the notion that the DNA duplication we described here comprises a fundamental mechanism in bacterial genome evolution.

## Results and Discussion

### *de novo* tandem repeats (TRs) in the β-lactamase gene that confer the extended substrate spectrum

**Figure 1:**
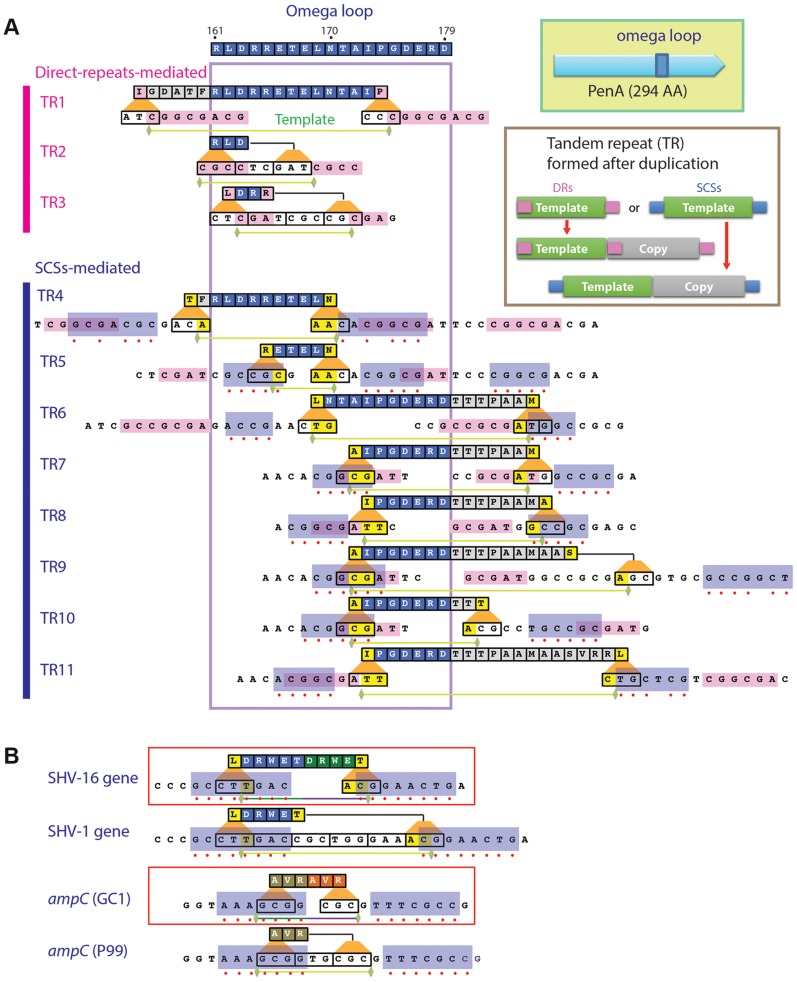
Local duplications in β-lactamase PenA. A. Map of the peptides subjected to duplication and pairs of small nucleotide sequences that apparently mediated the underlying DNA duplication (formation of TRs). The entire omega loop is displayed at the top with the amino acids denoted using the one-letter codes and positions numbered according to Ambler *et al.* [47]. Direct repeats or same-strand complementary sequences (SCSs) associated with each DNA template are denoted by pink or blue boxes, respectively, and matching positions between the pair of SCSs are denoted with red dots. Green horizontal bars underneath denote the ends of the DNA templates. B. SCSs associated with the TRs in β-lactamase genes from clinical isolates. The SCSs identified in two β-lactamase genes with TR mutations, SHV-16 gene from *K. pneumoniae* and *ampC* from *Enterobacter cloacae* GC1, are shown in boxes. Below of each of the genes are their parental alleles, the SHV-1 gene and *ampC* in *E. cloacae* strain P99, respectively. The paired upstream and downstream elements are denoted in blue boxes.

To determine if TRs can be formed naturally in β-lactamases under antibiotic pressure, we conducted a selection experiment by exposing *Burkholderia thailandensis* [11] to ceftazidime (3–5 µg/ml). The resistant colonies were screened for isolates with variants of the *penA* gene that acquired a duplication within the coding region (Figure 1A). PenA is a class A β-lactamase (BTH_II1450 from *B. thailandensis* strain E264) that confers resistance to amoxicillin. We observed that the relative frequency of occurrence of the TR mutations compared to substitution mutations was about 1 to 50. The frequency for substitution mutations was previously estimated to be 10^−8^ to 10^−7^ [12]. PenA has been used to explore evolutionary paths by various mutations to substrate spectrum extension [12], [13]. PenA from *B. thailandensis* is highly conserved in pathogenic *Burkholderia* species including *Burkholderia pseudomallei*, *Burkholderia mallei*, and *Burkholderia cenocepacia* [14]–[16]. The antibiotic regimen used to treat infections by these *Burkholderia* pathogens generally includes ceftazidime [17].

The *de novo* TRs in *penA* from the ceftazidime-resistant isolates, which we named TR1 through TR11, consisted of two repeat units (the template and the duplicate) that involve at least part of the region encoding the omega loop (Figure 1A). Alterations of the omega loop caused by specific mutations have been implicated in substrate spectrum extension [18], [19]. In a study in which a variable five amino acid cassette was randomly introduced into TEM-1, all insertions that conferred enhanced resistance to ceftazidime were consistently found at various positions within the omega loop [20].

The lengths of the repeat units of these TRs varied widely ranging from 9 bp (TR2 and TR3, coding for 3 amino acids) to 60 bp (TR11, coding for 20 amino acids). Except for TR2, all TRs had a discrepancy between the repeat units and the reading frame (codons) in the gene, and this often resulted in a different amino acid in the first position of the repeated peptide (Figure 1A).

### All TRs were associated with a pair of specific sequences

We discovered that three of the TRs (those numbered 1 to 3) were associated with a pair of direct repeats (Figure 1A; for nucleotide sequences of TRs and a comparison before and after duplication, see Figure S1). Although the lengths varied from 3 to 8, all had a common pattern in that the direct repeats were perfectly-matching to each other and the DNA template for duplication starts with the upstream repeat and ends just before the downstream repeat (Figure 1A, Figure S1). It is reasonable to assume that DNA duplication specifically defined by these direct repeats is mediated by the replication slippage mechanism established in various genomes [2].

We found that the other group of TRs (those numbered 4 to 11) also had direct repeats but these repeats were not bordering the template as in TRs 1 to 3, obscuring their role in DNA duplication (Figure 1A). In addition to the direct repeats, these TRs had a different duo of sequences (Figure 1A, Figure S1). These sequences had an interesting pattern—each pair had a high degree of seeming complementarity (Figure 1A). However, these sequences were on the same strand, and so we named them SCSs (same-strand complementary sequences). Whereas five TRs (5, 6, 7, 8, and 10) had perfectly matching SCSs, others had the ones with mismatches (Figure 1A).

Besides the mismatches, SCSs also exhibited variations in their positions relative to the template for TRs (Figure 1A). There undoubtedly would be selective pressure for a template during DNA duplication. The minimum requirement would be to maintain the reading frame in the gene with a multiple of 3 nucleotides in the repeat unit. Then the next selection requirement would be steric modification of the active site to accommodate new drugs without inducing serious protein structural instability.

### SCSs-mediated TR formation is common across β-lactamase types and bacterial groups

We found SCSs around TRs in the two clinical isolates obtained to date: in the SHV-16 gene from *K. pneumoniae* [5] and in the *ampC* gene, coding for a class C β-lactamase, from *E. cloacae* GC1 [7] (Figure 1B). The SCSs in these genes were perfectly matching, in contrast to those with occasional mismatches in *penA* in *B. thailandensis* (Figure 1A). No direct repeats were found around the DNA template in these genes, suggesting that the SCSs in TRs 4 to 11 may be functionally sufficient in mediating DNA duplication. These β-lactamase genes from distinct sources comprise the evidence supporting the identity of SCSs and that DNA duplication mediated by SCSs is a common mechanism for the substrate spectrum extension across β-lactamase types and bacterial groups.

### Point mutations affecting seven SCSs altered the TR patterns in *penA*

**Figure 2:**
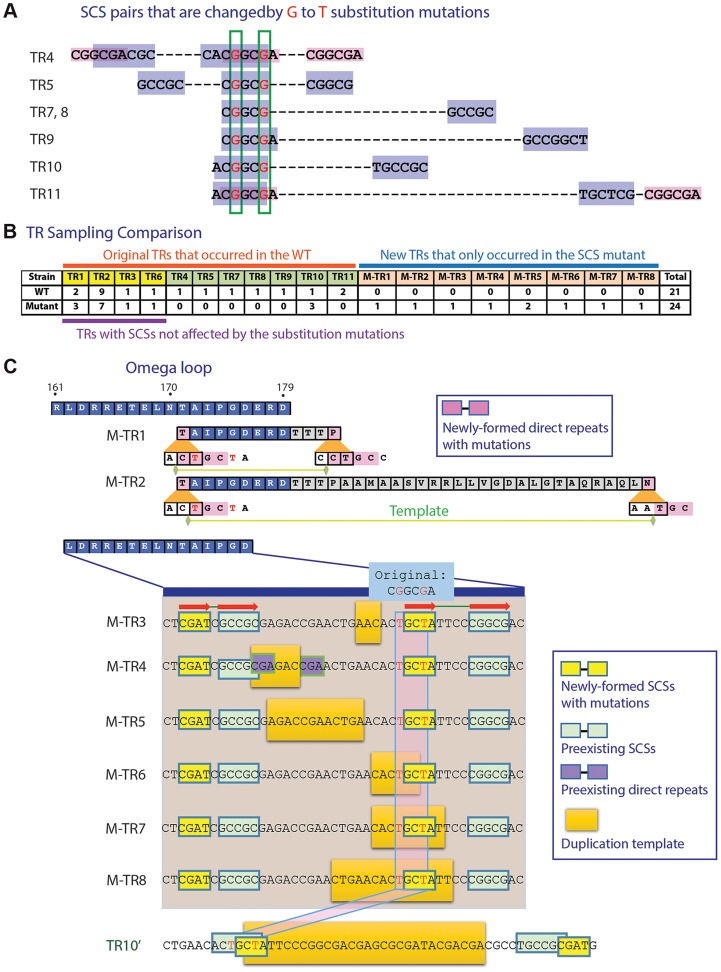
Mutation study with an SCS element. A. Two G to T substitution mutations introduced into a region overlapping seven SCS elements. Positions subjected to substitution mutations are shown in red. B. TR formation patterns compared between the wild type and the mutated *penA*. C. TRs formed in the mutated *penA* gene. M-TRs 1 to 8 that were newly formed in the mutated gene and TR10′, a version of TR10 in the wild type gene (Figure 1A), are shown. Common SCSs for M-TRs 3 to 8 are denoted with red arrows.

To test if SCSs mediate TR formation, we generated two point mutations in the wild-type *penA* in the region corresponding to the 3′-ends of the DNA templates for TR4 and TR5, in the way not affecting the encoded amino acids (see Materials and Methods, Figure 2A). This region also overlaps with the region at the 5′-ends of the templates for TRs 7 to 11 (Figure 2A). Therefore, the substitution mutations can alter the seven pairs of SCSs (Figure 2A). Against the selection with ceftazidime, the *penA* gene with the disrupted SCSs exhibited a similar TR pattern to that of the wild type in the region not containing the mutations (that for TRs 1, 2, 3, and 6) (Figure 2B; for principal components analysis (PCA) plots [21] comparing the wild type and the mutant, see Figure S2A). However, a significantly altered pattern was observed around the point mutations in that six of the seven TRs were not formed in the mutated gene (Figure 2B, Figure S2A). The only one that was still formed in the mutant was TR10 (Figure 2B). However, this formation of TR10 in the mutated gene may have been enabled by a new pair of SCSs generated around the DNA template, complementing the disrupted original SCS pairs (Figure 2C).

In addition, eight new TRs were formed in the mutated gene (Figure 2C; for a comparison between before and after duplication, see Figure S2B). Two of these, M-TR1 and M-TR2, occurred via direct repeats made with the new T nucleotides, demonstrating the mechanistic role of direct repeats specifically bordering the DNA template as in TRs 1 to 3 (Figure 1A). Likewise, M-TRs 3 to 8 apparently were formed by a pair of common SCSs made with the new T's along with a pair of neighboring SCSs, providing a basis for strong SCS interactions in the region (Figure 2C). These M-TRs may demonstrate the functional role of SCSs in DNA duplication, and also that different regions can be used as templates during DNA duplication mediated by the same set of SCSs. In fact, TR7 and TR8 were also resulted from duplication of slightly different template areas mediated by the same set of SCSs (Figure 1A). In the case of M-TR4, however, a short pair of direct repeats with TR1-type positioning was also present with the possibility of the alternative mechanism (Figure 2C). Together, the altered TR patterns in the SCS-altered gene strongly support the active role of SCSs as well as direct repeats with TR1-type positioning in DNA duplication in the β-lactamase gene.

### Various duplicates conferred a range of the ceftazidime-hydrolyzing activity

**Figure 3:**
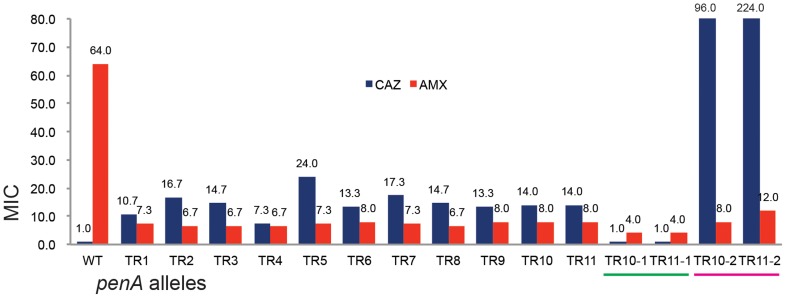
The MIC levels of the TR mutants. The MIC levels for ceftazidime (CAZ) and ampicillin (AMP) are shown in the bar graph. WT is the wild type *B. thailandensis* E264; TRs 1 to 11 are, E264 (*penA*-TRs 1 to 11); TR10-1 or TR11-1 are, E264 (Δ*penA*-TR10 or TR11); TR10-2 or TR11-2 are, E264 (Δ*penA*-TR10 or TR11, complemented with a plasmid carrying the undisrupted gene).

The minimum inhibitory concentration (MIC) for ceftazidime of the TR mutants were in the range of 7.3 (TR4) to 24 (TR5) (Figure 3). Significant correlation between the MICs and the size or the location of the duplication was observed (Figure 3). When the *penA* gene with TR10 or TR11 (*penA*-TR10 or *penA*-TR11, respectively, representing the TR-containing genes) was disrupted (see Materials and Methods), the strain lost resistance to ceftazidime (MIC≤1.0 µg/ml), having an MIC comparable to the level of the wild-type strain with Δ*penA* (Figure 3). When these strains were provided with an intact copy of the *penA*-TR10 or *penA*-TR11 *in trans*, ceftazidime resistance was restored (Figure 3), demonstrating that *penA*-TRs were the sole factors responsible for ceftazidime resistance. It is notable that resistance to ceftazidime can be dramatically increased through increased expression of the β-lactamase gene either when the gene is placed on a multicopy plasmid (Figure 3) or by a point mutation in the promoter of the gene [12], [22].

Substrate spectrum extension by mutations in β-lactamases is typically accompanied by a loss of the catalytic activity in the original substrates [8]. Accordingly, we observed decreased levels of MICs with one of the original antibiotic, amoxicillin (Figure 3). Three additional antibiotics (cefotaxime, ceftriaxone, and cefepime) that could be hydrolyzed by the wild-type enzyme [12] were tested with the bacteria harboring either of the two *penA*-TRs (*penA*-TR10 and *penA*-TR11) (Table 1). These strains also exhibited decreased levels of resistance to these original substrates, as has been observed with many class A β-lactamase mutants with acquired activity against third-generation cephalosporins [23]. In addition, the hydrolytic activities against amoxicillin were effectively inhibited by a β-lactamase inhibitor clavulanic acid (Table 1).

**Table 1:** MICs for various β-lactams with *B. thailandensis* strains.

Strain	MIC (µg/ml)^a^			
	AMX/CLA	CTXM	CRX	CEF
**Wild type**				
E264 (*penA*-WT)	6	6	16	18
**Mutant**				
E264 (*penA*-TR10)	3	1.5	1	8
E264 (*penA*-TR11)	3	1.625	0.5	12
***penA*** **-null mutant strains**				
E264 (Δ*penA*-WT)	1.5	1	0.5	3
E264 (Δ*penA*-TR10)	2	0.75	0.25	6
E264 (Δ*penA*-TR11)	2	1	0.38	6
***penA*** **-null mutants complemented by ** ***penA*** ** alleles carried by pRK415K**				
E264 (Δ*penA*-WT, complemented)	48	>32	>32	64
E264 (Δ*penA*-TR10, complemented)	2.5	3	2	24
E264 (Δ*penA*-TR11, complemented)	3	4.5	4	40

*^a^*MICs were measured by the E-test.

Abbreviations: AMX, amoxicillin; CLA, clavulanic acid; CTXM, cefotaxime; CRX, ceftriaxone; CEF, cefepime.

### The reversion of TRs via general cellular pathways

**Figure 4:**
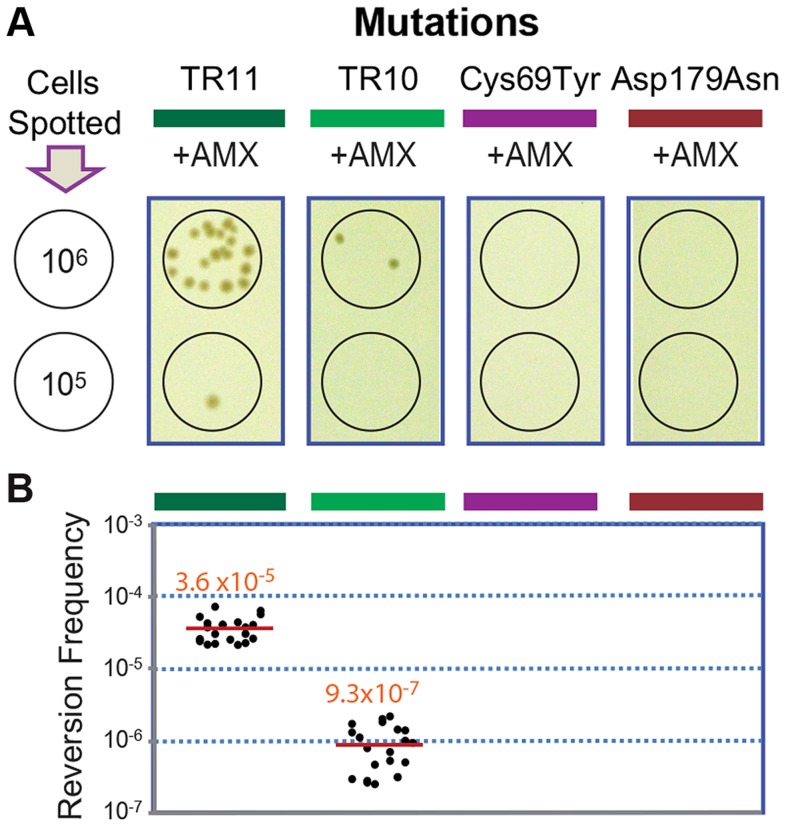
Reversion assays with strains carrying different *penA* alleles. A. Reversion assays. The strains with the *penA* mutations TR11, TR10, Cys69Tyr, and Asp179Asn were tested for their ability to undergo reversion. Serial dilutions of cells were spotted on selection plates containing amoxicillin (AMX), and spots of 10^6^ or 10^5^ cells from which revertant colonies arose (from the TR mutants) are shown. B. Reversion frequencies. Each measurement was plotted, and the mean value is denoted by a red line.

The original and the extended substrate spectrums of a β-lactamase define two phases of distinct catalytic activity [8], [24]. The reversion back to the wild type of most mutations including point mutations is highly unlikely at typical mutation rates, especially when the associated fitness cost is not significant [25]. Accordingly, we failed to observe reversion in *penA* with the point mutations Cys69Tyr and Asp179Asn [12] under selection against amoxicillin at a concentration that was tolerated by the wild-type strain but not by the mutants (16 µg/ml, Figure 4). In contrast, when the strains carrying *penA*-TR10 or *penA*-TR11 were challenged with the same antibiotic pressure, revertants were observed (Figure 4A). The average reversion frequency of the strain carrying *penA*-TR11 with a longer repeat was 3.6×10^−5^, which was more than ten-fold higher than that of the strain carrying *penA*-TR10 (9.3×10^−7^, Figure 4B).

The reversion capacity of the TR mutants of β-lactamase genes may be based on the intrinsic instability of TRs in genomes, as has been previously reported in various bacteria and eukaryotes including humans [1], [3], [26]. The instability in TRs in bacterial genomes is based on general cellular pathways including replication, recombination, and DNA repair systems [2], [4], [26]. Bacterial cellular pathways (recombination in particular) have been the focus of attention for alternative drug targets to prevent the generation and spread of antibiotic resistance [27], [28]. The TR-based antibiotic resistance further lends support to such approaches in the battle against bacterial antibiotic resistance.

### The omega loop has high potential for DNA duplication with high contents of SCSs and direct repeats

**Figure 5:**
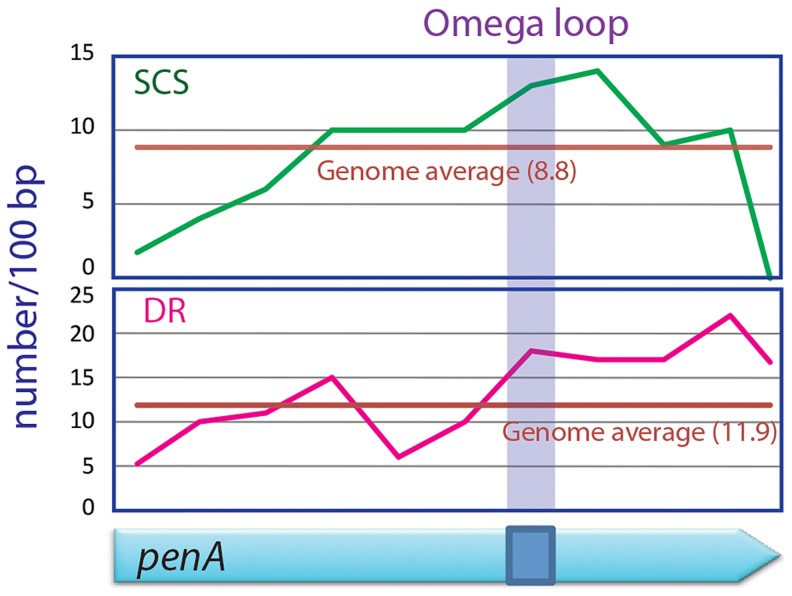
SCSs and direct repeats contents in the omega loop compared with the rest of the *penA* gene and the genome of *B. thailandensis*. The region encoding the omega loop has high contents for SCSs and direct repeats (DR).

We scanned the *penA* gene and the entire genome of *B. thailandensis* for direct repeats and SCSs, and found that the region encoding the omega loop in *penA* has unusually high contents of both sequences (Figure 5). It was particularly distinct with SCSs in that the omega loop and the immediate downstream region had the highest number for the sequences. These data suggest that there is high DNA duplication potential in the omega loop region, and support the significance of the region as the hotbed for the evolution of the β-lactamase. This omega loop region with a high potential of mutations makes the β-lactamase gene a so called “contingency gene” that facilitates the efficient exploration of phenotypic solutions to unpredictable (host) environment [3].

### DNA duplication mediated by direct repeats and SCSs comprises a fundamental mechanism shaping the bacterial evolution

**Figure 6:**
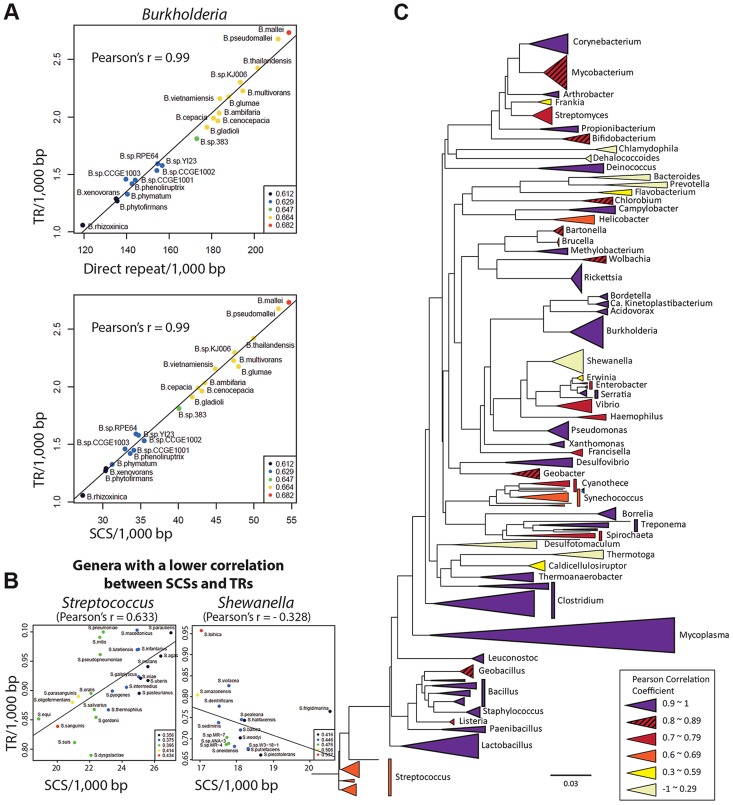
Genome-level correlation analysis between TRs and SCSs or direct repeats. A. TRs-direct repeats and TRs-SCSs correlations in *Burkholderia*. A high Pearson correlation coefficient of 0.99 was measured in both analyses, suggesting that the formation of TRs are largely attributable to both direct repeats and SCSs in *Burkholderia*. B. SCSs-TRs correlations of two genera representing mid and low levels (*Streptococcus* and *Shewanella*, respectively). The entire set of the data is shown in Figure S3. C. The SCSs-TRs correlation profile in the bacterial phylogenetic tree. Bacterial genera are color-coded based on the levels of the Pearson correlation coefficients.

As direct repeats have been implicated in DNA duplication at the genomic level [2], a high correlation is expected between the direct repeats content and DNA duplication activities in genomes. Accordingly, we observed a high Pearson correlation coefficient (0.9) between the number of direct repeats and that of TRs among the *Burkholderia* spp.—the hosts for the *penA* gene (Figure 6A). We also measured a high correlation (Pearson correlation coefficient of 0.99) between the number of SCSs and that of TRs across genomes (Figure 6A). This suggests that SCSs are also associated with DNA duplication activities in *Burkholderia* at the genomic level, and that DNA duplication mediated by both direct repeats and SCSs comprise a general mechanism in *Burkholderia*.

Then we expanded the analysis to include diverse bacterial genomes. All completed bacterial genomes were downloaded from the National Center for Biotechnology Information (NCBI) and 1,387 genomes (those having the largest genome in a species) were selected as representatives for each species. These genomes were grouped in genera, and the ones with at least five members (species), which were a total of 59 genera, were subjected to the analysis. In the analysis comparing the contents of SCSs and TRs among the members within each genus, we found 42 genera with at least 0.7 of the Pearson correlation coefficient, including 25 genera with a highly significant correlation (>0.9) (Figure S3). However, there were also the genera with poor correlations (Figure 6B; Figure S3). Genera with similar correlation coefficients, especially distinctive with the low ones, showed a tendency to form clusters in the phylogenetic tree, and the GC contents in the genomes had no significant correlation with such patterns (Figure 6C). This phylogenetic profile could be interpreted as partial or total loss of the SCSs-mediated DNA duplication mechanism in some bacterial groups during the course of the evolution. An analysis with direct repeats also revealed the distribution of the high correlation with TR levels among the most bacterial genera, with lower correlations observed in some groups (Figure S3). Together, the direct repeats-TRs and SCSs-TRs correlation patterns in the bacterial kingdom suggest that DNA duplication mediated by direct repeats and SCSs comprises a fundamental mechanism shaping bacterial genome evolution.

### Perspectives

**Figure 7:**
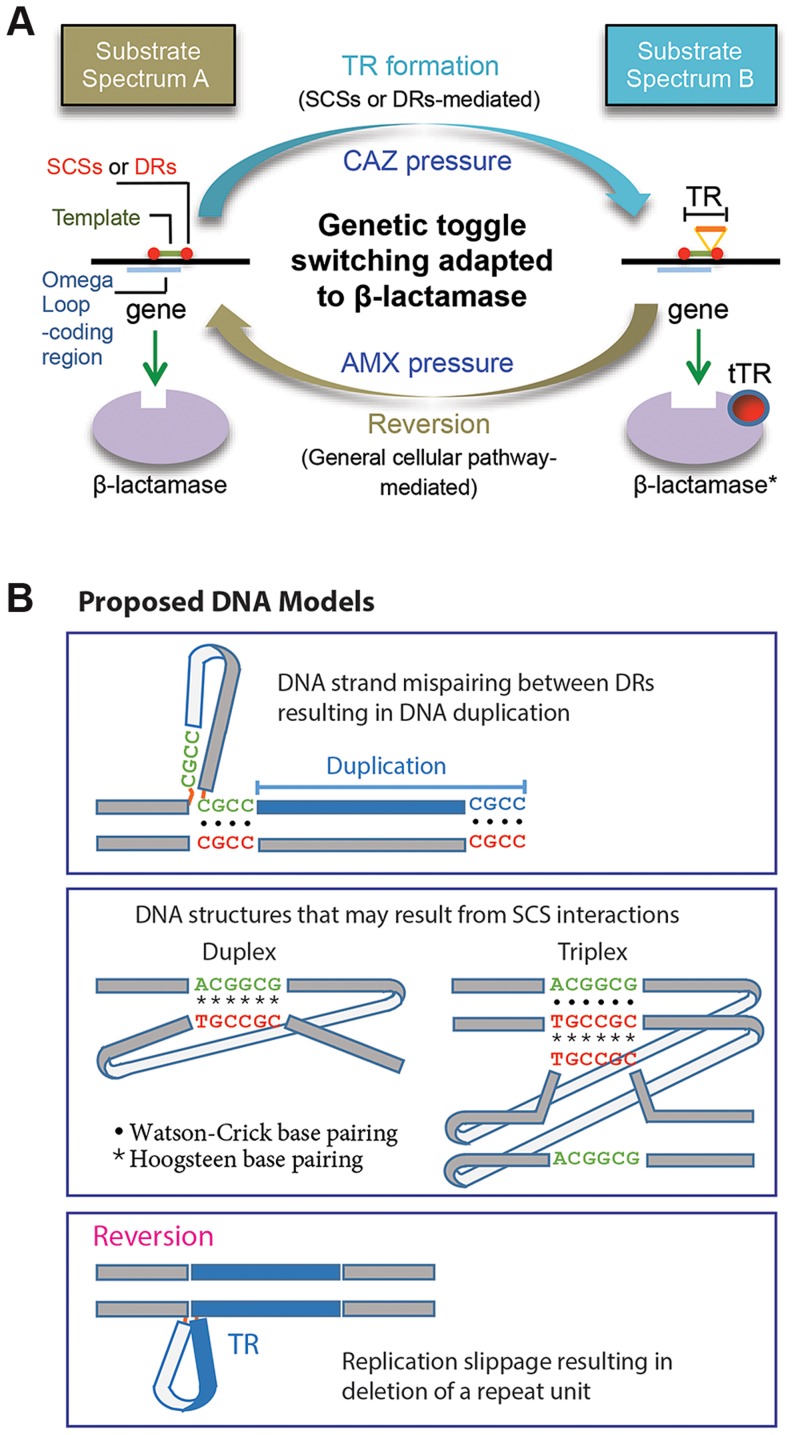
Proposed models. A. A model for the DNA duplication-reversion cycle serving as a genetic toggling mechanism for the β-lactamase substrate spectrum. Same-strand complementary sequences (SCSs)- or direct repeat-mediated DNA duplication can be selected for in a β-lactamase gene by exposure to ceftazidime (CAZ). The resulting tandem repeat (TR) in the omega loop encodes a duplicated peptide, which results in an alteration in the active site cavity extending the substrate spectrum to include CAZ (substrate spectrum B). The β-lactamase gene with a TR mutation can be reverted to the wild type by exposure to an original antibiotic, amoxicillin (AMX) (substrate spectrum A). The reversion of the TR does not require direct repeats or SCSs but is likely mediated by the replication slippage mechanism, involving general cellular pathway. B. Models for DNA secondary structures. A model is shown for mispaired direct repeats (DRs) that can occur during DNA replication, leading to DNA duplication of the bordered DNA template. TRs 1 to 3 (Fig. 1A) and M-TRs 1 to 2 (Fig. 2C) may have resulted from this mechanism. Two models of DNA structures that may form through non-canonical base pairings between SCS elements. Such DNA structures may predispose to DNA duplication via an unknown mechanism. Lastly, a model is shown for a DNA structure formed by strand slippage during replication, which leads to reversion.

In this study we characterized the formation of DNA duplication in the coding region of a β-lactamase gene. The *de novo* TRs, coupled with reversion, has been adapted to mediate reversible switching between the two states of the β-lactamase substrate spectrums for bacterial survival against dynamic antibiotic challenges (Figure 7A). A characteristic attribute of this novel toggling mechanism is its function on enzyme activities. This is in contrast with the toggle switch constructed with genes arranged in a mutually inhibitory network, by which transcription of the genes is alternated [29]. As a practical sense, the TR-based toggles may have implications for biotechnology for the use to reversibly affect the activity of a target protein.

The direct repeats at the specific positions relative to the DNA template—as those in TRs 1 to 3 and M-TRs 1 and 2—fit perfectly to the known mechanism, DNA replication slippage [2] (Figure 7B). By contrast, the role of the direct repeats without such positioning—as those in TRs 4 to 11—is not clear. Unlike direct or inverted repeats, SCSs would not interact with each other through usual Watson-Crick base pairing. This suggests that a novel form of DNA conformation would be involved during this DNA duplication, possibly through reverse Watson-Crick or reverse Hoogsteen base pairing [30]–[33] (Figure 7B). Non-canonical base interactions have been found more prevalent in DNA than previously predicted, and in some DNA sequences Hoogsteen base pairs are in thermal equilibrium with Watson-Crick base pairs [34]. If such SCSs-based DNA structures are formed (Figure 7B), facilitated by unknown factors and/or by certain local topological strain, they may cause structural instability in the region, causing mistakes in the DNA replication, repair, or recombination machineries, predisposing to DNA duplication [26], [30].

Although the underlying mechanism is not understood, the biological consequences resulting from the SCSs-mediated DNA duplication are significant. In this study, we showed that more TRs were mediated by SCSs than by direct repeats in the β-lactamase gene, *penA*, during selection (Figure 1A, Figure 2ABC). That SCS-TRs may be common across different types of β-lactamases (the classes A and C) and bacterial groups (Figure 1B), and that SCSs may be involved in more fundamental processes shaping the bacterial evolution (Figure 6) signifies the importance of SCSs. It is intriguing to note that sequences corresponding to SCSs were also found over-represented in the flanking regions of insertion and deletion mutations in various eukaryote genomes [35], and association of such sequences with human genetic diseases was suggested [36]. The potential pivotal role of SCSs in the evolution of both prokaryotic and eukaryotic genomes and in human diseases further assure the need for the active investigations on SCSs.

## Materials and Methods

### Bacterial cultures

All *Escherichia coli* strains were grown in Luria Bertani (LB) media, and all *B. thailandensis* strains were grown in LB or AB minimal media containing 0.25% glucose (ABG) [37] at 37°C. The concentrations of antibiotics used for *E. coli* were as follows: tetracycline, 10 µg/ml; kanamycin, 50 µg/ml; and ampicillin, 100 µg/ml. For *B. thailandensis*, the concentrations of tetracycline and kanamycin used were 50 µg/ml and 250 µg/ml, respectively.

### Characterization of the mutations in *penA*

To map the mutations in *penA* (BTH_II1450 in the strain E264 genome), we began by sequencing the gene. Genomic DNA from each ceftazidime-resistant mutant was purified using a Wizard Genomic DNA Purification Kit (Promega, Madison, WI, USA) and was used as the template for the PCR amplification of *penA* and its short flanking regions to yield a 1,386-bp amplicon (271 bp upstream from the start codon of the gene to 230 bp downstream of the stop codon). PCR reactions were conducted in a 50-µl reaction mixture containing 2.5 U of HotStar HiFidelity polymerase (Qiagen, Hilden, Germany), 50 pmol of the primers penA-F (5′-CGTCAATCCGATGCAGTACC-3′) and penA-R (5′-GCCGTTATCGCACCTTTATC-3′), 100 ng of template DNA, 10 µl of 5× Q Solution, and 10 µl of 5× HotStar HiFidelity buffer. The reaction protocol consisted of an initial enzyme-activating step (95°C for 5 min), an amplification step (35 cycles at 94°C for 15 sec, 61°C for 1 min, and 72°C for 1.4 min), and a final extension step (72°C for 10 min). Gel-purified 1.4 kb PCR products were sequenced using a 3730XL DNA analyzer (Applied Biosystems, Foster City, CA, USA) in both directions using the primers penA-F and penA-R.

### Determination of the MIC (minimal inhibitory concentration) values

The MIC values were measured using the E-test [38], following the manufacturer's instructions (AB Biodisk, Solna, Sweden). Briefly, each strain was grown on Müller-Hinton agar plates at 37°C for 2 days. For the strains harboring pRK415K-derived plasmids, Müller-Hinton agar supplemented with kanamycin (250 µg/ml) was used. Single colonies from the plates were suspended in 2 ml of 0.85% NaCl until the turbidity reached the 0.5 McFarland standard. Using sterile cotton swabs, the cell suspensions were spread on Müller-Hinton agar plates, the E-test strips were placed, and the plates were incubated at 37°C for 16–18 h. Using the E-test strips, the lowest concentration where no visible growths was observed was recorded as the MIC. The average values were calculated from triplicate experiments.

The agar dilution method was conducted as described by Wiegand *et al.* [39]. Briefly, a single colony of each strain grown on Müller-Hinton agar was used to inoculate 2 ml of Müller-Hinton broth, and the culture was incubated overnight with shaking (250 rpm) at 37°C. The overnight cultures were serially diluted with fresh Müller-Hinton broth and dispensed into the wells of a 96-well microtiter plate. Using a multi-channel micropipette, 1 µl of the diluted bacterial suspensions was placed on each Müller-Hinton agar plate containing ceftazidime at various concentrations and incubated at 37°C for 16 h. The lowest concentration of antibiotic such that there was no visible bacterial growth was observed in the spot containing approximately 10^4^ CFU (colony forming unit), which was recorded as the MIC. The number of CFUs in serially diluted bacterial suspensions was determined by spreading 100 µl of the appropriately diluted bacterial suspensions on LB agar plates, followed by incubating the plates for 24 h at 37°C, and counting the colonies derived from viable cells. The average values were calculated from triplicate experiments.

### Construction of *penA*-null mutants

The wild-type and mutated *penA* genes were disrupted as follows. First, the wild-type *penA* was PCR-amplified using the primer pairs penA-KF (5′-ATATATGGTACCCGTCAATCCGATGCAGTACC-3′) and penA-KR (5′-ATATATGGTACCGCCGTTATCGCACCTTTATC-3′) that contained a *Kpn*I recognition site (underlined) at the end. The 1.4-kb PCR product was then digested with *Kpn*I and ligated into pUC19, which was digested using the same enzyme. The resulting plasmid was double-digested with *Xho*I and *Pfl*FI to remove an 195-bp internal region (position 124 bp to 319 bp) in *penA*, blunt ended with T4 DNA polymerase (NEB, Ipswich, MA, USA), and inserted with a tetracycline-resistance (*tet* ^r^) cassette by ligation. The *tet* ^r^ cassette was previously amplified from pRK415K [40] using HotStar HiFidelity polymerase (Qiagen, Hilden, Germany) and primers tetR-F (5′-ATATATCTCGAGGTGAGGCTTGGACGCTAGG-3′) and tetR-R (5′-ATATTTCTCGAGCTTGGATCAGACGCTGAGTG-3′) that contained an *Xho*I recognition site (underlined), *Xho*I-digested, and blunt-ended with T4 DNA polymerase. The construct was transferred into *B. thailandensis* strains employing a modified method of natural transformation [41]. Briefly, 3 ml of a defined medium (DM) that was prepared as described by Thongdee *et al.* [41] was inoculated with a single colony freshly grown on LB agar and then incubated overnight with shaking at 250 rpm at 37°C. The 200-µl overnight culture was diluted with 10 ml of fresh preheated DM, and the culture was grown with shaking at 250 rpm at 37°C to an OD_600_ of approximately 0.5. The culture was then concentrated 20-fold in 500 µl of fresh DM and 50 µl aliquots of the concentrated cells were mixed with 0.5 µg of plasmid DNA. The mixture was incubated for 30 min on ice, and 2 ml of DM preheated to 37°C was added before the mixture was incubated overnight at 250 rpm at 37°C. After washing the culture with 1 ml of fresh DM and resuspending the pellet in 250 µl of fresh DM, 100 µl of the cell suspension was plated on ABG medium containing 50 µg/ml tetracycline and incubated at 37°C for 48 hrs to select for *tet* ^r^ cassette-containing constructs. An obtained *penA* null mutant was verified by PCR using the primer pair of penA_LF (5′-AACAGATCGCCGAGATGG-3′) and penA_LR (5′-GCGAACGTTGCCCGATAC-3′), which hybridizes to the genomic regions outside the DNA sequence that is used for mutant construction.

### Complementation of the *penA* mutations

*Kpn*I-treated PCR products of mutated *penA*, which was amplified using the primers penA-KF and penA-KR as described above, were ligated with *Kpn*I-treated pRK415K. This plasmid was transferred into the *E. coli* strain S17-1 [42] using a conventional transformation method [43]. The transformed S17-1 strain was conjugated with a *B. thailandensis penA*-null mutant on ABG agar plates containing 250 µg/ml kanamycin, and the plates were incubated at 37°C for 2 days to select for transconjugants. A successful conjugation was confirmed by purifying the plasmid from the transconjugants and examining the characteristic restriction patterns of the plasmid.

### Reversion frequency analysis

To estimate the reversion frequency of the mutations in *penA*, we compared the colony-forming units of the serially diluted strains on LB plates without (for total cell counts) and with amoxicillin at a final concentration of 16 µg/ml (for revertant counts). The alleles of *penA* in the revertants were sequenced for verification. Each of the serially diluted strains was prepared as follows. First, each of the strains was grown on an LB agar plate for two days, and a colony was then suspended in LB at a concentration of approximately 2×10^5^/µl. This cell suspension was then serially diluted in LB. Five microliters of the cell suspension and 5 µl of each dilution were spotted on LB plates, and the plates were incubated for approximately 24 to 30 hours until colonies formed.

### Construction of the strain with *penA* gene mutations in a common SCS element

The strain with *penA* gene with two substitution mutations in a common SCS element (CACGGCG to CACTGCT) (Figure 2A) was constructed as follows. The DNA fragment of 1,445 bp, which spans the whole *penA*-TR11 gene and the extra flanking sequences was constructed by ligating two separately prepared fragments. The first half of the gene, which spans from the position 271 bp upstream to the position 527 bp downstream from the start codon, was obtained using PCR conducted in a 50 µl reaction mixture containing 1 U of KOD FX NEO polymerase (TOYOBO, Japan), 15 pmol each of the primers penA-KF (5′-ATATATGGTACCCGTCAATCCGATGCAGTACC-3′), containing a *Kpn*I recognition site (underlined), and penA-R-527 (5′-GTGTTCAGTTCGGTCTCGCG-3′), 100 ng of the genomic DNA from the strain containing *penA*-TR11, 25 µl of 2× PCR buffer for KOD FX Neo and 10 µl of 2 mM dNTPs. The reaction consisted of the following three steps: initial pre-denaturation step (94°C for 2 min), amplification step (35 cycles of 98°C for 10 sec, 64°C for 1 min, and 68°C for 48 sec), and the final extension step (72°C for 10 min). The second half of the gene spans from the position 528 bp downstream from the start codon to the position 229 bp downstream from the stop codon. This fragment was amplified as described above except for using a different pair of primers: penA-T2-528 (5′-TGCTATTCCCGGCGACGAGC-3′), in which two point mutations (underlined) were introduced and penA-ER (5′-ATATATGAATTCGCCGTTATCGCACCTTTATCGC-3′), which contains an *EcoR*I recognition site (underlined). The resulting two fragments were phosphorylated with polynucleotide kinase (NEB, Ipswich, MA, USA) and were digested with *Kpn*I and *EcoR*I, respectively, and the fragments were cloned into *Kpn*I and *EcoR*I digested pUC19. The ligated *penA*-TR11 gene with introduced substituted mutations was cut out of the pUC19 construct by digesting the construct with *Kpn*I and *EcoR*I, and the gene fragment was blunt-ended with T4 DNA polymerase (NEB, Ipswich, MA, USA). Then the gene fragment was cloned into a suicide vector, pSR47S [44]. The resulting pSR47S construct was introduced into *Burkholderia thailandensis* E264 using triparental mating. Briefly, the recipient strain *Burkholderia thailandensis* E264 was grown in LB overnight, and the donor strain *E. coli* DH5αλpir containing pSR47S construct and the helper strain *E. coli* containing pRK600 were grown overnight in LB supplemented with kanamycin at 50 µg/ml and chloramphenicol at 20 µg/ml, respectively. The overnight cultures of the three strains were washed with PBS and were mixed to the final volume of 100 µl, and the mixture was spread on an LB agar plate and was incubated at 37°C for 3 hours. Then the cells were resuspended in 2 ml of PBS and were spread on an LB agar plate supplemented with ceftazidime at 5 µg/ml and 5% sucrose and were incubated for 48 hours at 37°C. The obtained strain containing the allele-exchanged *penA* gene with substitution mutations was plated on an LB agar plate supplemented with amoxicillin at 16 µg/ml, to select for a revertant, which has the *penA* gene with two substitution mutations. Allele exchange and reversion were verified by carrying out PCR with primers penA_OF (5′-AACAGATCGCCGAGATGG-3′) and penA_OR(5′-GCGAACGTTGCCCGATAC-3′), which anneal to the regions outside the cloned region, and by sequencing the PCR product.

### Analysis of SCSs, direct repeats, and TRs in bacterial genomes

Bacterial genomes were downloaded from the National Center for Biotechnology Information (NCBI). For simpler computations, SCSs were confined to those with perfect complementarity with a minimum unit size of 6 bps and a distance between the units of 6 to 100 bps. Direct repeats were confined to those with a minimum unit size of 5 bps and have a distance between the repeats of 6 to 100 bps. Customized perl scripts were used to identify the SCSs and direct repeats in genomes. For TRs, only those with the unit length equal to or larger than 6 bases (capacity for a peptide with two amino acids) were counted. TRs were identified using the software tool Tandem Repeat Finder [45].

To construct the phylogenetic tree with selected bacterial genera, 16S rDNA sequences of the selected genera were aligned using PyNAST [46]. Gaps were removed using a Qiime script (http://www.qiime.com), and then the tree was built using clustalX2 (http://www.clustal.org/clustal2/).

## Supporting Information

Figure S1 Local sequence duplication in the wild-type *penA* gene. The DNA sequences corresponding to the TRs listed in Figure 1A are provided.
(PDF)

Figure S2 Mutation study with an SCS element. A. CA (correspondence analysis) plots of the wild-type and the mutant *penA* gene using their TR-formation patterns. In the first CA plot, the wild type (WT-1) and the mutant (M-1) are at the same position when only the frequencies of the TRs not affected by the substitution mutations (TRs 1, 2, 3, and 6) are used. However, the wild type and the mutant (marked as WT-2 and M-2, respectively) are distinctive based on the other TRs (TRs 4, 5, 7, 8, 9, 10, and 11) and M-TRs, the occurrence of which is affected by the substitution mutations in the gene. In the second CA plot, individual mutations are plotted. All mutations fall into three groups, except TR10. B. The sequences of M-TRs and TR10′ with and without duplication as listed in Figure 2C.
(PDF)

Figure S3 SCSs-TRs and direct repeats-TRs correlations in bacterial genera. A. SCSs-TRs correlations and GC contents in bacterial genera. B. SCSs-TRs correlation in each bacterial genus. C. Direct repeats-TRs correlation in each bacterial genus.
(PDF)
