# Supplementary material for: The Tandem Repeats Enabling Reversible Switching between the Two Phases of β-Lactamase Substrate Spectrum
Source: PLoS Genet. 2014 Sep 18;10(9):e1004640. doi: 10.1371/journal.pgen.1004640 (PMC4169377; doi:10.1371/journal.pgen.1004640)

Figure S1. Local sequence duplication in the wild-type *penA* gene. The DNA sequences corresponding to the TRs listed in Fig. 1A are provided.

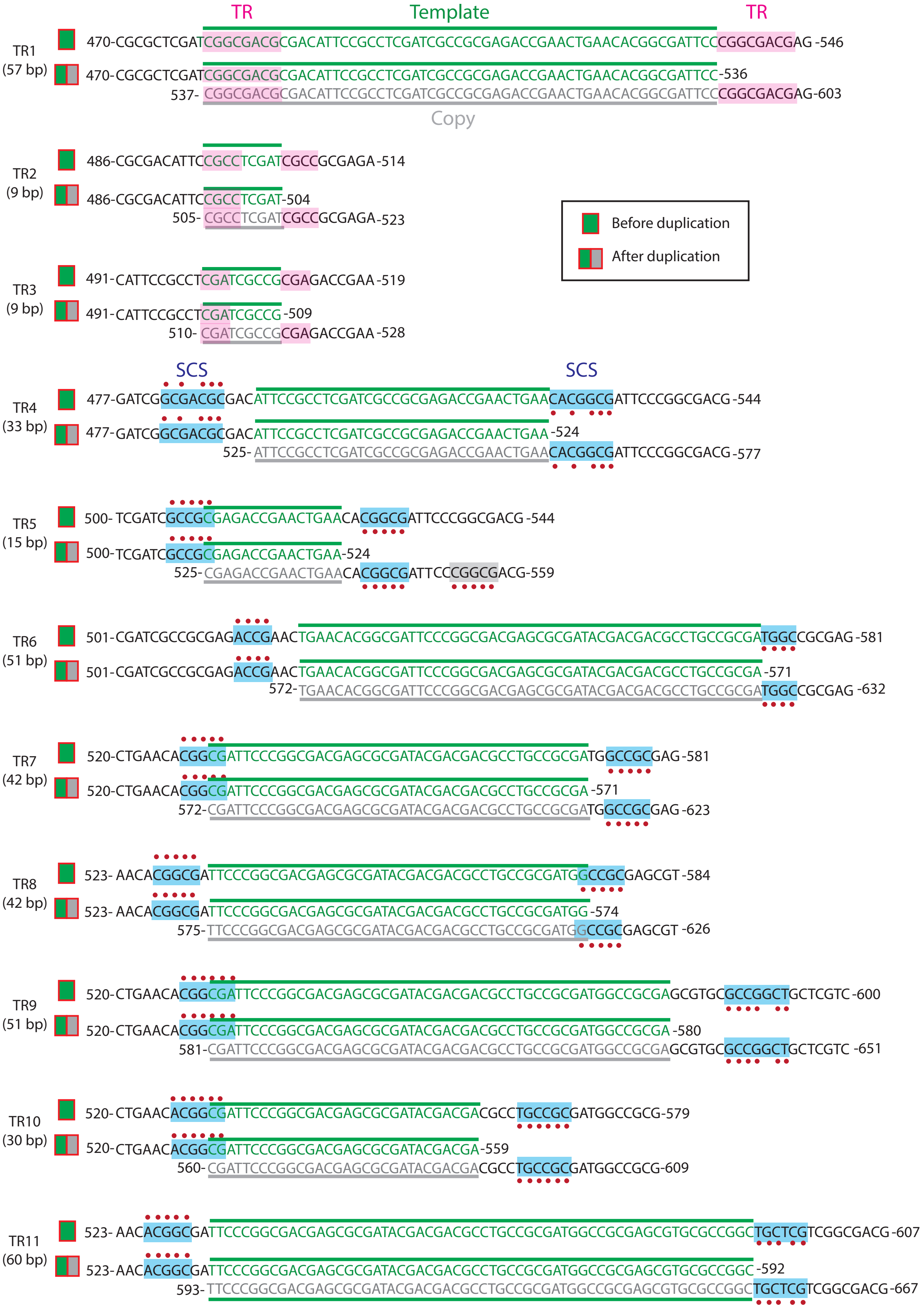

Supplement: Figure S1 — Local sequence duplication in the wild-type penA gene. The DNA sequences corresponding to the TRs listed in Figure 1A are provided. (PDF) [file pgen.1004640.s001.pdf]
