# Supplementary material for: The Tandem Repeats Enabling Reversible Switching between the Two Phases of β-Lactamase Substrate Spectrum
Source: PLoS Genet. 2014 Sep 18;10(9):e1004640. doi: 10.1371/journal.pgen.1004640 (PMC4169377; doi:10.1371/journal.pgen.1004640)

Figure S2. Mutation study with an SCS element.

A. CA (correspondence analysis) plots of the wild-type and the mutant *penA* gene using their TR-formation patterns. In the first CA plot [1], the wild type (WT-1) and the mutant (M-1) are at the same position when only the frequencies of the TRs not affected by the substitution mutations (TRs 1, 2, 3, and 6) are used. However, the wild type and the mutant (marked as WT-2 and M-2, respectively) are distinctive based on the other TRs (TRs 4, 5, 7, 8, 9, 10, and 11) and M-TRs, the occurrence of which is affected by the substitution mutations in the gene. In the second CA plot, individual mutations are plotted. All mutations fall into three groups, except TR10.

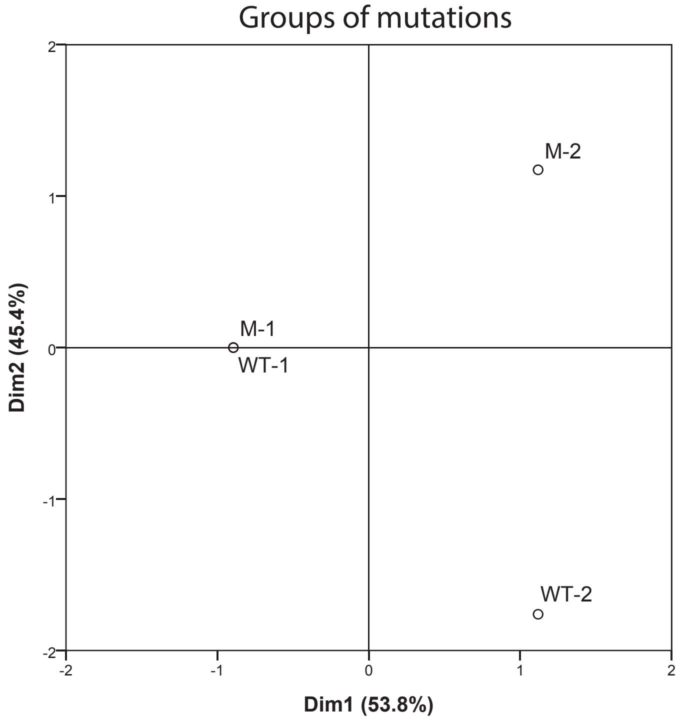

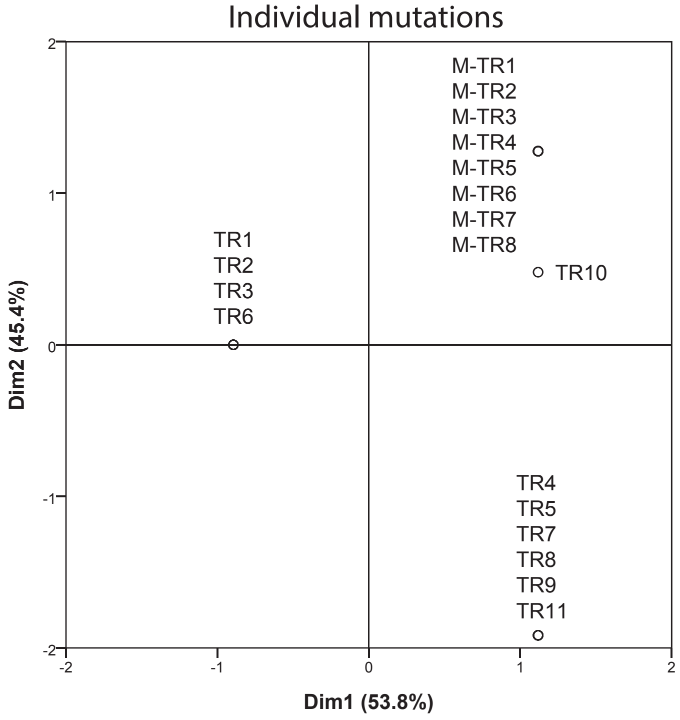

Reference

1. Hirschfeld HO. A connection between correlation and contingency; 1935. Cambridge Univ Press. pp. 520-524.

B. The sequences of M-TRs and TR10′ with and without duplication as listed in Fig. 2C.

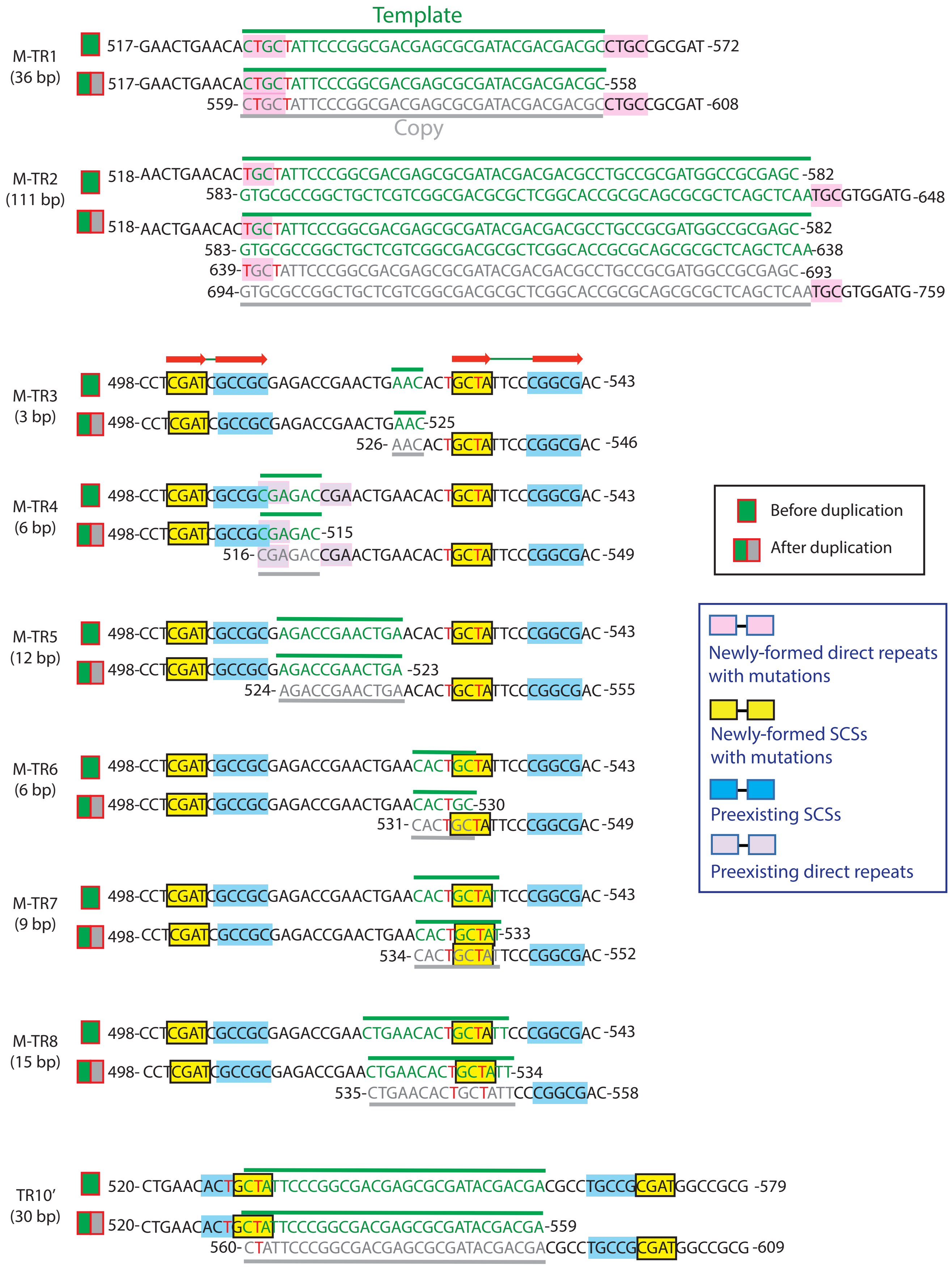

Supplement: Figure S2 — Mutation study with an SCS element. A. CA (correspondence analysis) plots of the wild-type and the mutant penA gene using their TR-formation patterns. In the first CA plot, the wild type (WT-1) and the mutant (M-1) are at the same position when only the frequencies of the TRs not affected by the substitution mutations (TRs 1, 2, 3, and 6) are used. However, the wild type and the mutant (marked as WT-2 and M-2, respectively) are distinctive based on the other TRs (TRs 4, 5, 7, 8, 9, 10, and 11) and M-TRs, the occurrence of which is affected by the substitution mutations in the gene. In the second CA plot, individual mutations are plotted. All mutations fall into three groups, except TR10. B. The sequences of M-TRs and TR10′ with and without duplication as listed in Figure 2C. (PDF) [file pgen.1004640.s002.pdf]
