# Supplementary material for: The Tandem Repeats Enabling Reversible Switching between the Two Phases of β-Lactamase Substrate Spectrum
Source: PLoS Genet. 2014 Sep 18;10(9):e1004640. doi: 10.1371/journal.pgen.1004640 (PMC4169377; doi:10.1371/journal.pgen.1004640)
Supplement: Figure S3 — SCSs-TRs and direct repeats-TRs correlations in bacterial genera. A. SCSs-TRs correlations and GC contents in bacterial genera. B. SCSs-TRs correlation in each bacterial genus. C. Direct repeats-TRs correlation in each bacterial genus. (PDF) [file pgen.1004640.s003.pdf]

Figure S3. SCSs-TRs and direct repeats-TRs correlations in bacterial genera

A. SCSs-TRs correlations and the GC contents in bacterial genera

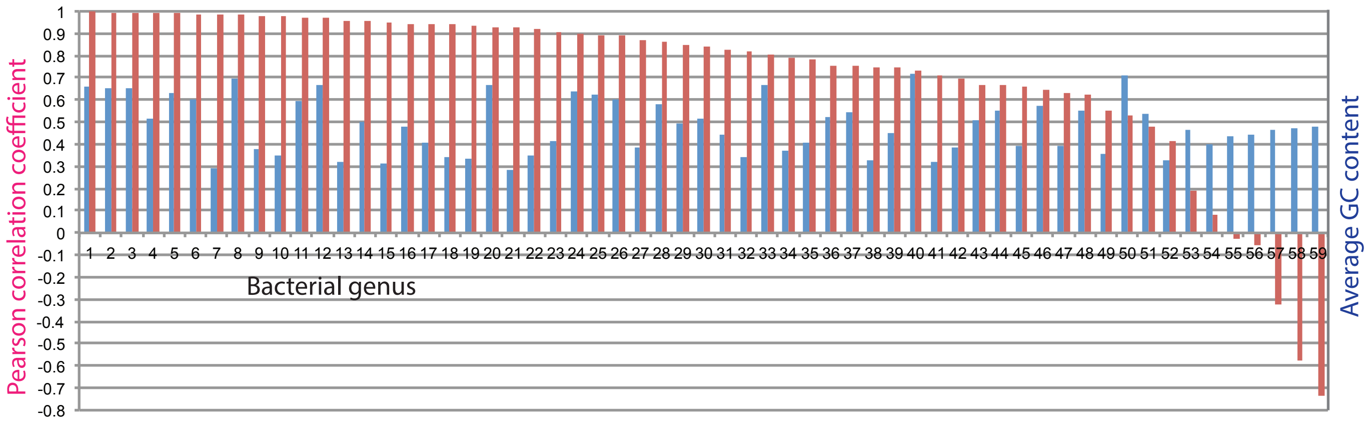

Bacterial genus

1	Bordetella	11	Corynebacterium	21	Borrelia
2	Propionibacterium	12	Acidovorax	22	Thermoanaerobacte
3	Burkholderia	13	Rickettsia	23	Lactobacillus
4	Serratia	14	Paenibacillus	24	Xanthomonas
5	Arthrobacter	15	Candidatus Kinetoplastibacteriu	25	Pseudomonas
6	Desulfovibrio	16	Treponema	26	Bifidobacterium
7	Mycoplasma	17	Bacillus	27	Bartonella
8	Methylobacterium	18	Clostridium	28	Geobacter
9	Leuconostoc	19	Staphylococcus	29	Geobacillus
10	Campylobacter	20	Deinococcus	30	Chlorobium

31	Pyrococcus	41	Methanocaldococcus	51	Erwinia
32	Wolbachia	42	Haemophilus	52	Flavobacterium
33	Mycobacterium	43	Thermococcus	53	Desulfotomaculum
34	Listeria	44	Synechococcus	54	Chlamydophila
35	Cyanothece	45	Helicobacter	55	Bacteroides
36	Spirochaeta	46	Brucella	56	Thermotoga
37	Enterobacter	47	Streptococcus	57	Shewanella
38	Francisella	48	Pyrobaculum	58	Dehalococcoides
39	Vibrio	49	Caldicellulosiruptor	59	Prevotella
40	Streptomyces	50	Frankia		

B. SCSs-TRs correlation in each bacterial genus

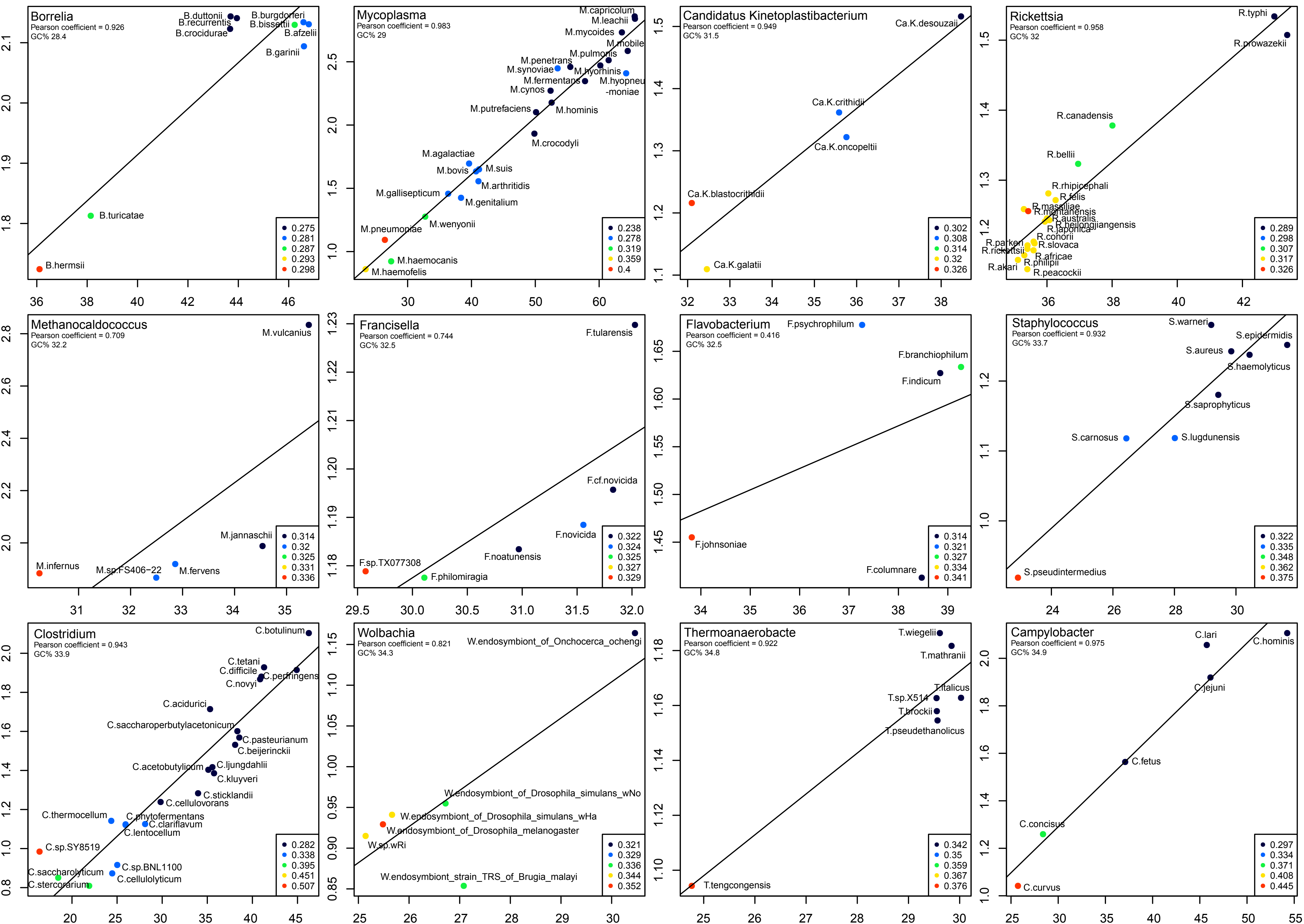

Borrelia
Pearson coefficient = 0.926
GC% 28.4
B.duttonii
B.recurrentis
B.crocidurae
B.burgdorferi
B.bissettii
B.afzelii
B.garinii
B.turicatae
B.hermsii
0.275
0.281
0.287
0.293
0.298
Mycoplasma
Pearson coefficient = 0.983
GC% 29
M.capricolum
M.leachii
M.mycoides
M.mobile
M.pulmonis
M.penetrans
M.synoviae
M.hyorhinis
M.fermentans
M.hyopneu-moniae
M.cynos
M.putrefaciens
M.hominis
M.crocodyli
M.agalactiae
M.bovis
M.suis
M.arthritidis
M.gallisepticum
M.genitalium
M.wenyonii
M.pneumoniae
M.haemocanis
M.haemofelis
0.238
0.278
0.319
0.359
0.4
Candidatus Kinetoplastibacterium
Pearson coefficient = 0.949
GC% 31.5
Ca.K.desouzaii
Ca.K.crithidii
Ca.K.oncopeltii
Ca.K.blastocrithidii
Ca.K.galatii
0.302
0.308
0.314
0.32
0.326
Rickettsia
Pearson coefficient = 0.958
GC% 32
R.typhi
R.prowazekii
R.canadensis
R.bellii
R.rhipicephali
R.felis
R.massiliae
R.montanensis
R.australis
R.heilongjiangensis
R.japonica
R.conorii
R.parkeri
R.slovaca
R.rickettsii
R.africae
R.philipii
R.akari
R.peacockii
0.289
0.298
0.307
0.317
0.326
Methanocaldococcus
Pearson coefficient = 0.709
GC% 32.2
M.vulcanius
M.jannaschii
M.infernus
M.sp.FS406−22
M.fervens
0.314
0.32
0.325
0.331
0.336
Francisella
Pearson coefficient = 0.744
GC% 32.5
F.tularensis
F.cf.novicida
F.novicida
F.noatunensis
F.sp.TX077308
F.philomiragia
0.322
0.324
0.325
0.327
0.329
Flavobacterium
Pearson coefficient = 0.416
GC% 32.5
F.psychrophilum
F.branchiophilum
F.indicum
F.johnsoniae
F.columnare
0.314
0.321
0.327
0.334
0.341
Staphylococcus
Pearson coefficient = 0.932
GC% 33.7
S.warneri
S.epidermidis
S.aureus
S.haemolyticus
S.saprophyticus
S.carnosus
S.lugdunensis
S.pseudintermedius
0.322
0.335
0.348
0.362
0.375
Clostridium
Pearson coefficient = 0.943
GC% 33.9
C.botulinum
C.tetani
C.difficile
C.perfringens
C.novyi
C.acidurici
C.saccharoperbutylacetonicum
C.pasteurianum
C.beijerinckii
C.acetobutylicum
C.ljungdahlii
C.kluyveri
C.sticklandii
C.cellulovorans
C.thermocellum
C.phytofermentans
C.clariflavum
C.lentocellum
C.sp.SY8519
C.sp.BNL1100
C.saccharolyticum
C.cellulolyticum
C.stercorarium
0.282
0.338
0.395
0.451
0.507
Wolbachia
Pearson coefficient = 0.821
GC% 34.3
W.endosymbiont_of_Onchocerca_ochengi
W.endosymbiont_of_Drosophila_simulans_wNo
W.endosymbiont_of_Drosophila_simulans_wHa
W.endosymbiont_of_Drosophila_melanogaster
W.sp.wRi
W.endosymbiont_strain_TRS_of_Brugia_malayi
0.321
0.329
0.336
0.344
0.352
Thermoanaerobacte
Pearson coefficient = 0.922
GC% 34.8
T.wiegelii
T.mathranii
T.italicus
T.sp.X514
T.brockii
T.pseudethanolicus
T.tengcongensis
0.342
0.35
0.359
0.367
0.376
Campylobacter
Pearson coefficient = 0.975
GC% 34.9
C.lari
C.hominis
C.jejuni
C.fetus
C.concisus
C.curvus
0.297
0.334
0.371
0.408
0.445

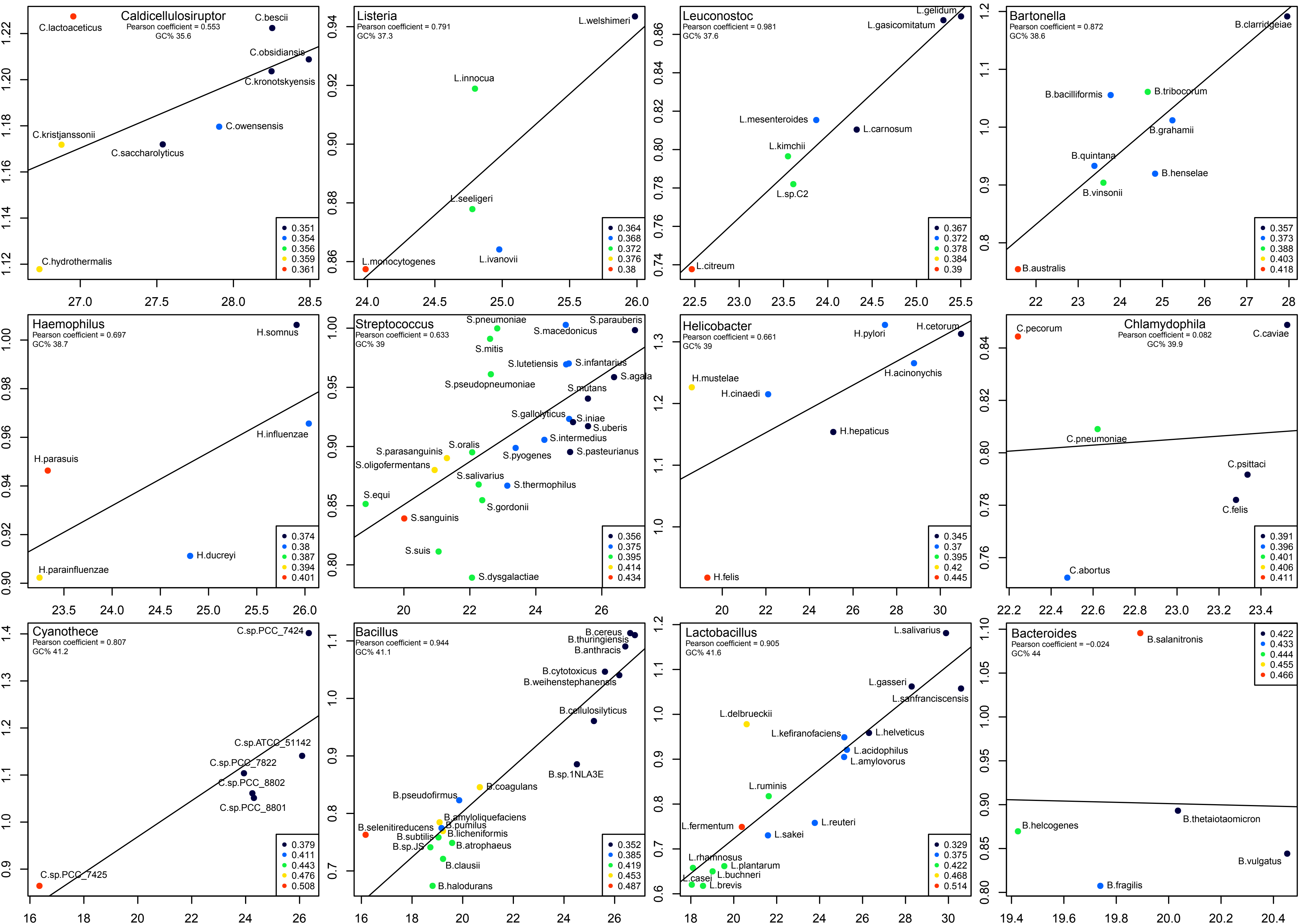
Caldicellulosiruptor
Pearson coefficient = 0.553
GC% 35.6
C.lactoaceticus
C.bescii
C.obsidiansis
C.kronotskyensis
C.owensensis
C.kristjanssonii
C.saccharolyticus
C.hydrothermalis
0.351
0.354
0.356
0.359
0.361
Listeria
Pearson coefficient = 0.791
GC% 37.3
L.welshimeri
L.innocua
L.seeligeri
L.ivanovii
L.monocytogenes
0.364
0.368
0.372
0.376
0.38
Leuconostoc
Pearson coefficient = 0.981
GC% 37.6
L.gelidum
L.gasicomitatum
L.mesenteroides
L.carnosum
L.kimchii
L.sp.C2
L.citreum
0.367
0.372
0.378
0.384
0.39
Bartonella
Pearson coefficient = 0.872
GC% 38.6
B.clarridgeiae
B.bacilliformis
B.tribocorum
B.grahamii
B.quintana
B.henselae
B.vinsonii
B.australis
0.357
0.373
0.388
0.403
0.418
Haemophilus
Pearson coefficient = 0.697
GC% 38.7
H.somnus
H.influenzae
H.parasuis
H.ducreyi
H.parainfluenzae
0.374
0.38
0.387
0.394
0.401
Streptococcus
Pearson coefficient = 0.633
GC% 39
S.pneumoniae
S.parauberis
S.macedonicus
S.mitis
S.lutetiensis
S.infantarius
S.agala
S.pseudopneumoniae
S.mutans
S.gallolyticus
S.iniae
S.uberis
S.intermedius
S.oralis
S.parasanguinis
S.pyogenes
S.pasteurianus
S.oligofermentans
S.salivarius
S.thermophilus
S.equi
S.gordonii
S.sanguinis
S.suis
S.dysgalactiae
0.356
0.375
0.395
0.414
0.434
Helicobacter
Pearson coefficient = 0.661
GC% 39
H.pylori
H.cetorum
H.acinonychis
H.mustelae
H.cinaedi
H.hepaticus
H.felis
0.345
0.37
0.395
0.42
0.445
Chlamydophila
Pearson coefficient = 0.082
GC% 39.9
C.pecorum
C.caviae
C.pneumoniae
C.psittaci
C.felis
C.abortus
0.391
0.396
0.401
0.406
0.411
Cyanothece
Pearson coefficient = 0.807
GC% 41.2
C.sp.PCC_7424
C.sp.ATCC_51142
C.sp.PCC_7822
C.sp.PCC_8802
C.sp.PCC_8801
C.sp.PCC_7425
0.379
0.411
0.443
0.476
0.508
Bacillus
Pearson coefficient = 0.944
GC% 41.1
B.cereus
B.thuringiensis
B.anthracis
B.cytotoxicus
B.weihenstephanensis
B.cellulosilyticus
B.sp.1NLA3E
B.coagulans
B.pseudofirmus
B.amyloliquefaciens
B.selenitireducens
B.pumilus
B.subtilis
B.licheniformis
B.sp.JS
B.atrophaeus
B.clausii
B.halodurans
0.352
0.385
0.419
0.453
0.487
Lactobacillus
Pearson coefficient = 0.905
GC% 41.6
L.salivarius
L.gasseri
L.sanfranciscensis
L.delbrueckii
L.kefiranofaciens
L.helveticus
L.acidophilus
L.amylovorus
L.ruminis
L.fermentum
L.reuteri
L.sakei
L.rhamnosus
L.plantarum
L.buchneri
L.casei
L.brevis
0.329
0.375
0.422
0.468
0.514
Bacteroides
Pearson coefficient = −0.024
GC% 44
B.salanitronis
B.thetaiotaomicron
B.helcogenes
B.vulgatus
B.fragilis
0.422
0.433
0.444
0.455
0.466

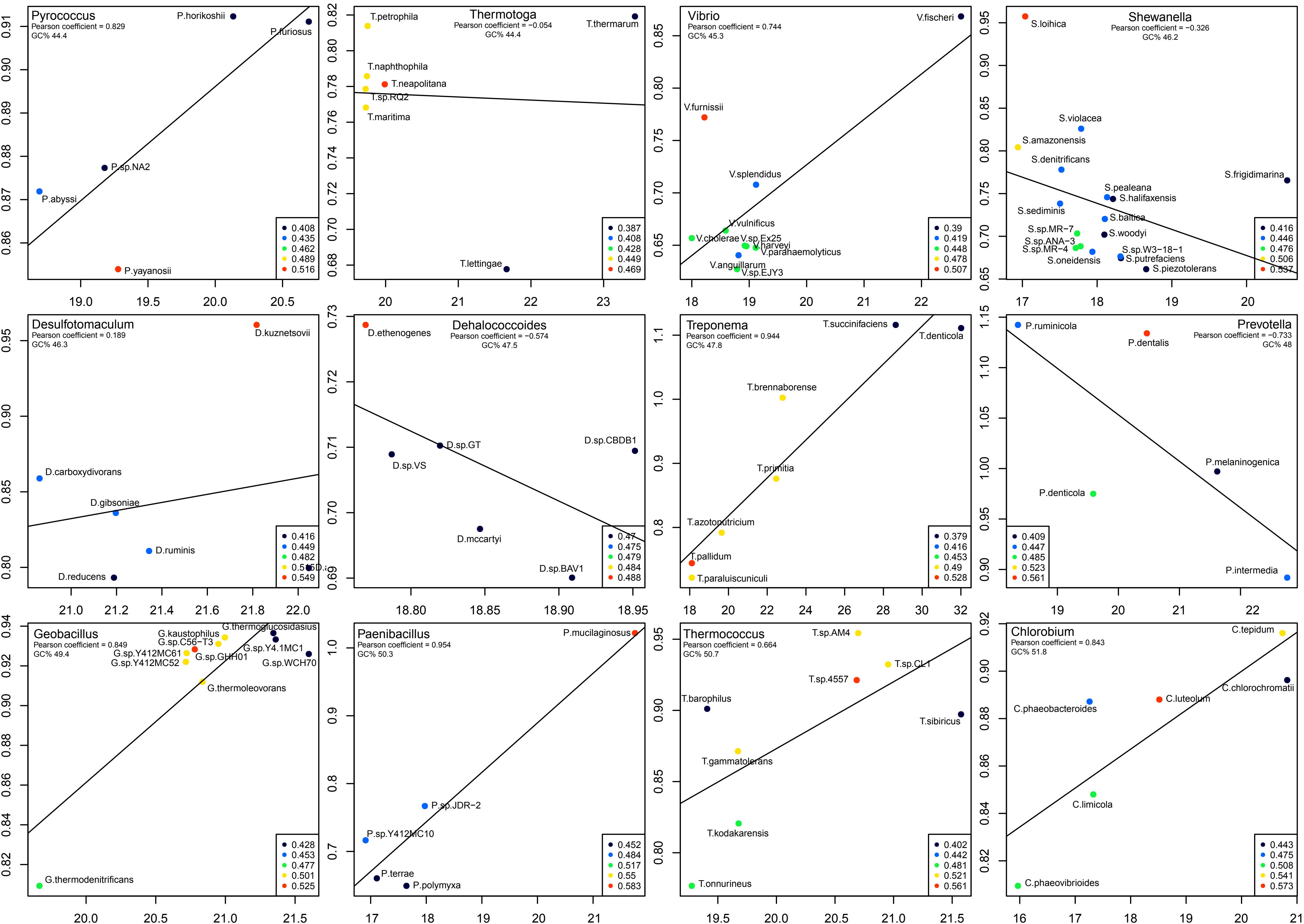

Pyrococcus
Pearson coefficient = 0.829
GC% 44.4
P.horikoshii
P.furiosus
P.sp.NA2
P.abyssi
P.yayanosii
0.408
0.435
0.462
0.489
0.516
Thermotoga
Pearson coefficient = −0.054
GC% 44.4
T.petrophila
T.thermarum
T.naphthophila
T.neapolitana
T.sp.RQ2
T.maritima
T.lettingae
0.387
0.408
0.428
0.449
0.469
Vibrio
Pearson coefficient = 0.744
GC% 45.3
V.fischeri
V.furnissii
V.splendidus
V.vulnificus
V.cholerae
V.sp.Ex25
V.harveyi
V.parahaemolyticus
V.anguillarum
V.sp.EJY3
0.39
0.419
0.448
0.478
0.507
Shewanella
Pearson coefficient = −0.326
GC% 46.2
S.loihica
S.violacea
S.amazonensis
S.denitrificans
S.frigidimarina
S.pealeana
S.halifaxensis
S.sediminis
S.baltica
S.sp.MR−7
S.woodyi
S.sp.ANA−3
S.sp.MR−4
S.sp.W3−18−1
S.putrefaciens
S.oneidensis
S.piezotolerans
0.416
0.446
0.476
0.506
0.537
Desulfotomaculum
Pearson coefficient = 0.189
GC% 46.3
D.kuznetsovii
D.carboxydivorans
D.gibsoniae
D.ruminis
D.reducens
0.416
0.449
0.482
0.515
0.549
Dehalococcoides
Pearson coefficient = −0.574
GC% 47.5
D.ethenogenes
D.sp.GT
D.sp.VS
D.sp.CBDB1
D.mccartyi
D.sp.BAV1
0.47
0.475
0.479
0.484
0.488
Treponema
Pearson coefficient = 0.944
GC% 47.8
T.succinifaciens
T.denticola
T.brennaborense
T.primitia
T.azotonutricium
T.pallidum
T.paraluiscuniculi
0.379
0.416
0.453
0.49
0.528
Prevotella
Pearson coefficient = −0.733
GC% 48
P.ruminicola
P.dentalis
P.melaninogenica
P.denticola
P.intermedia
0.409
0.447
0.485
0.523
0.561
Geobacillus
Pearson coefficient = 0.849
GC% 49.4
G.kaustophilus
G.thermoglucosidasius
G.sp.C56−T3
G.sp.Y4.1MC1
G.sp.Y412MC61
G.sp.GHH01
G.sp.Y412MC52
G.sp.WCH70
G.thermoleovorans
G.thermodenitrificans
0.428
0.453
0.477
0.501
0.525
Paenibacillus
Pearson coefficient = 0.954
GC% 50.3
P.mucilaginosus
P.sp.JDR−2
P.sp.Y412MC10
P.terrae
P.polymyxa
0.452
0.484
0.517
0.55
0.583
Thermococcus
Pearson coefficient = 0.664
GC% 50.7
T.sp.AM4
T.sp.CL1
T.sp.4557
T.barophilus
T.sibiricus
T.gammatolerans
T.kodakarensis
T.onnurineus
0.402
0.442
0.481
0.521
0.561
Chlorobium
Pearson coefficient = 0.843
GC% 51.8
C.tepidum
C.chlorochromatii
C.luteolum
C.phaeobacteroides
C.limicola
C.phaeovibrioides
0.443
0.475
0.508
0.541
0.573

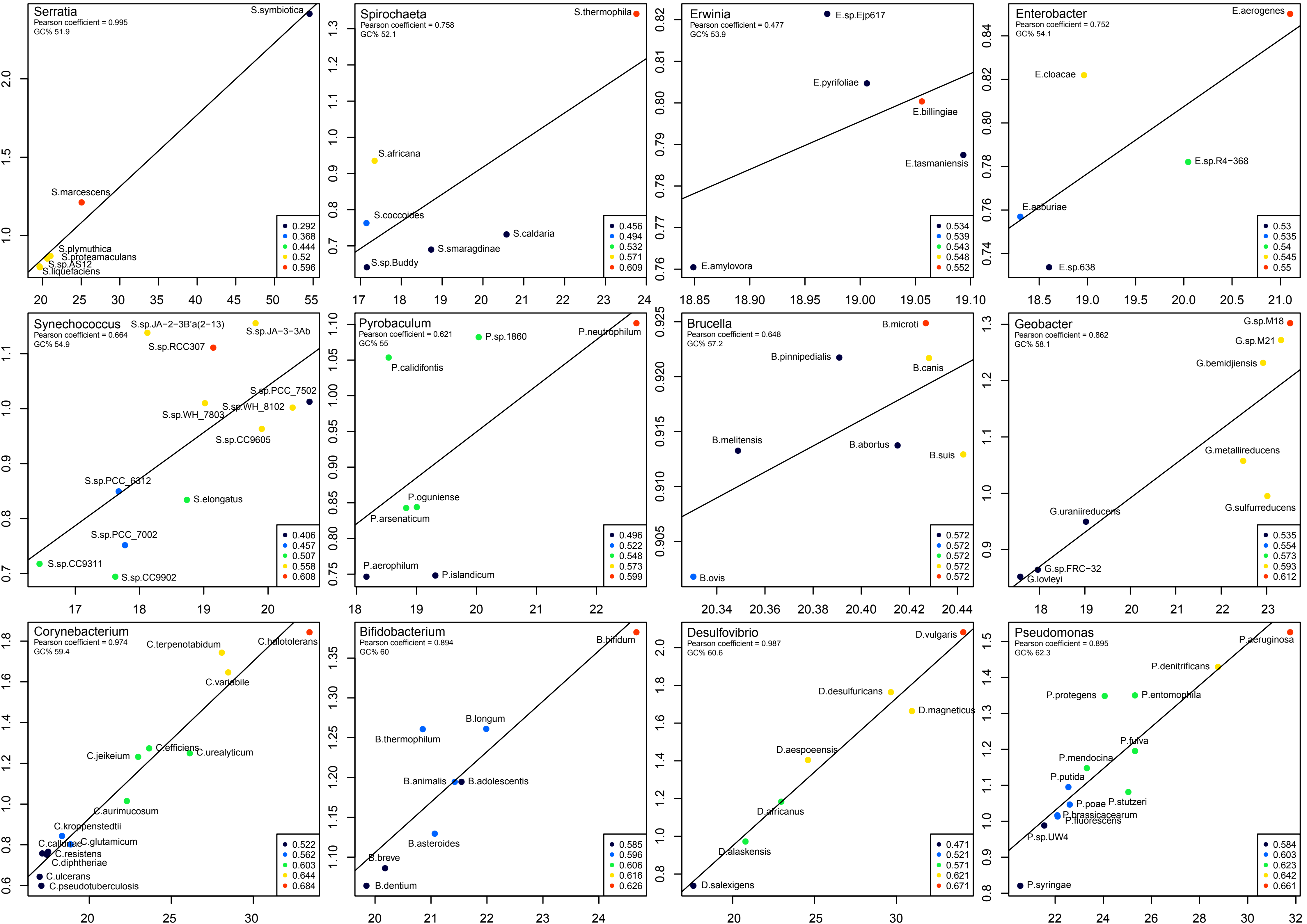
Serratia
Pearson coefficient = 0.995
GC% 51.9
Spirochaeta
Pearson coefficient = 0.758
GC% 52.1
Erwinia
Pearson coefficient = 0.477
GC% 53.9
Enterobacter
Pearson coefficient = 0.752
GC% 54.1
Synechococcus
Pearson coefficient = 0.664
GC% 54.9
Pyrobaculum
Pearson coefficient = 0.621
GC% 55
Brucella
Pearson coefficient = 0.648
GC% 57.2
Geobacter
Pearson coefficient = 0.862
GC% 58.1
Corynebacterium
Pearson coefficient = 0.974
GC% 59.4
Bifidobacterium
Pearson coefficient = 0.894
GC% 60
Desulfovibrio
Pearson coefficient = 0.987
GC% 60.6
Pseudomonas
Pearson coefficient = 0.895
GC% 62.3

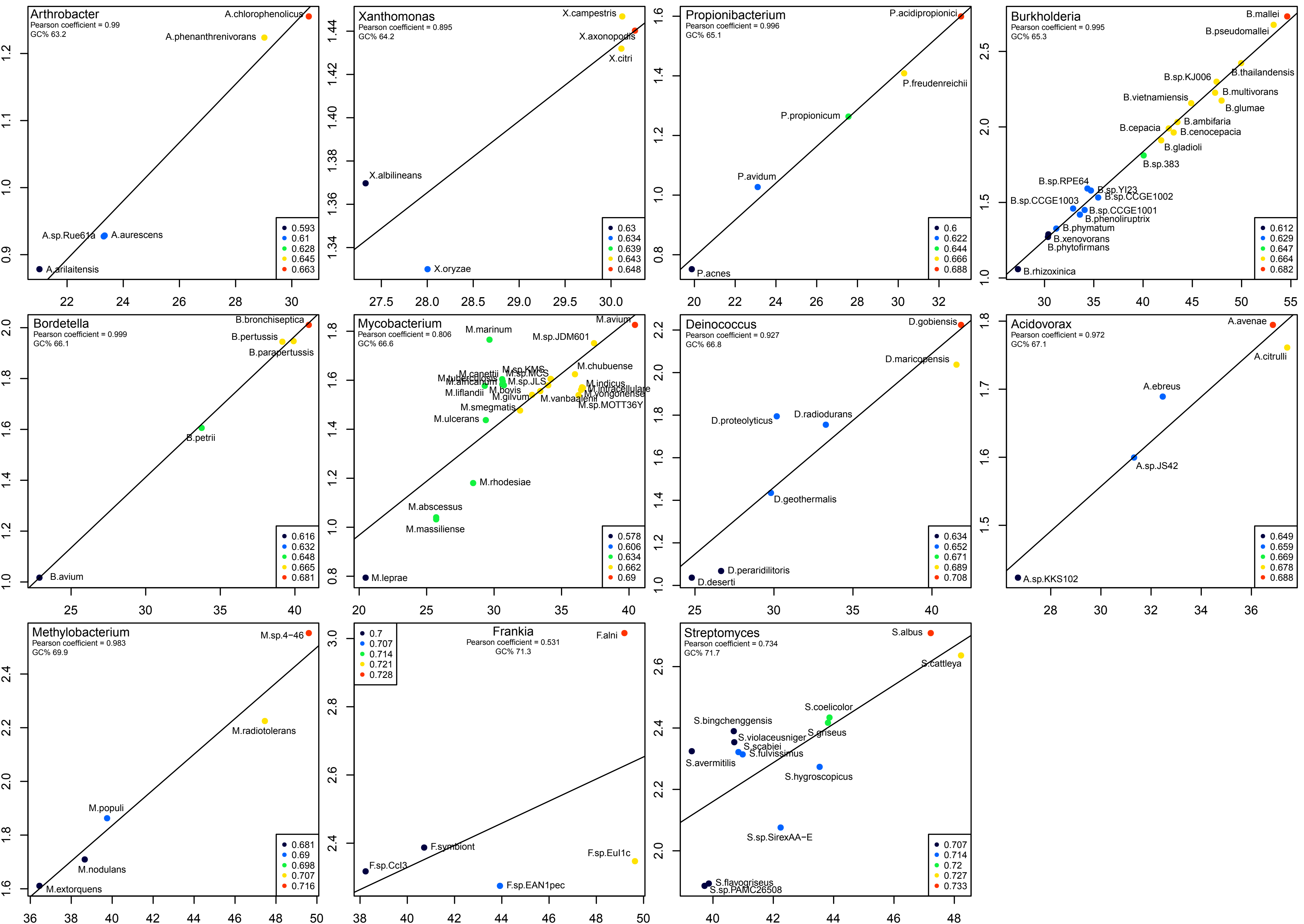

Arthrobacter
Pearson coefficient = 0.99
GC% 63.2
A.chlorophenolicus
A.phenanthrenivorans
A.sp.Rue61a
A.aurescens
A.arilaitensis
0.593
0.61
0.628
0.645
0.663
Xanthomonas
Pearson coefficient = 0.895
GC% 64.2
X.campestris
X.axonopodis
X.citri
X.albilineans
X.oryzae
0.63
0.634
0.639
0.643
0.648
Propionibacterium
Pearson coefficient = 0.996
GC% 65.1
P.acidipropionici
P.freudenreichii
P.propionicum
P.avidum
P.acnes
0.6
0.622
0.644
0.666
0.688
Burkholderia
Pearson coefficient = 0.995
GC% 65.3
B.mallei
B.pseudomallei
B.thailandensis
B.sp.KJ006
B.multivorans
B.vietnamiensis
B.glumae
B.ambifaria
B.cepacia
B.cenocepacia
B.gladioli
B.sp.383
B.sp.RPE64
B.sp.YI23
B.sp.CCGE1002
B.sp.CCGE1003
B.sp.CCGE1001
B.phenoliruptrix
B.phymatum
B.xenovorans
B.phytofirmans
B.rhizoxinica
0.612
0.629
0.647
0.664
0.682
Bordetella
Pearson coefficient = 0.999
GC% 66.1
B.bronchiseptica
B.pertussis
B.parapertussis
B.petrii
B.avium
0.616
0.632
0.648
0.665
0.681
Mycobacterium
Pearson coefficient = 0.806
GC% 66.6
M.avium
M.marinum
M.sp.JDM601
M.chubuense
M.sp.KMS
M.sp.MCS
M.canettii
M.tuberculosis
M.africanum
M.sp.JLS
M.bovis
M.liflandii
M.gilvum
M.indicus
M.intracellulare
M.yongonense
M.vanbaalenii
M.sp.MOTT36Y
M.smegmatis
M.ulcerans
M.rhodesiae
M.abscessus
M.massiliense
M.leprae
0.578
0.606
0.634
0.662
0.69
Deinococcus
Pearson coefficient = 0.927
GC% 66.8
D.gobiensis
D.maricopensis
D.radiodurans
D.proteolyticus
D.geothermalis
D.peraridilitoris
D.deserti
0.634
0.652
0.671
0.689
0.708
Acidovorax
Pearson coefficient = 0.972
GC% 67.1
A.avenae
A.citrulli
A.ebreus
A.sp.JS42
A.sp.KKS102
0.649
0.659
0.669
0.678
0.688
Methylobacterium
Pearson coefficient = 0.983
GC% 69.9
M.sp.4−46
M.radiotolerans
M.populi
M.nodulans
M.extorquens
0.681
0.69
0.698
0.707
0.716
Frankia
Pearson coefficient = 0.531
GC% 71.3
F.alni
F.symbiont
F.sp.CcI3
F.sp.EuI1c
F.sp.EAN1pec
0.7
0.707
0.714
0.721
0.728
Streptomyces
Pearson coefficient = 0.734
GC% 71.7
S.albus
S.cattleya
S.coelicolor
S.bingchenggensis
S.griseus
S.violaceusniger
S.scabiei
S.fulvissimus
S.avermitilis
S.hygroscopicus
S.sp.SirexAA−E
S.flavogriseus
S.sp.PAMC26508
0.707
0.714
0.72
0.727
0.733

C. Direct repeats-TRs correlation in each bacterial genus

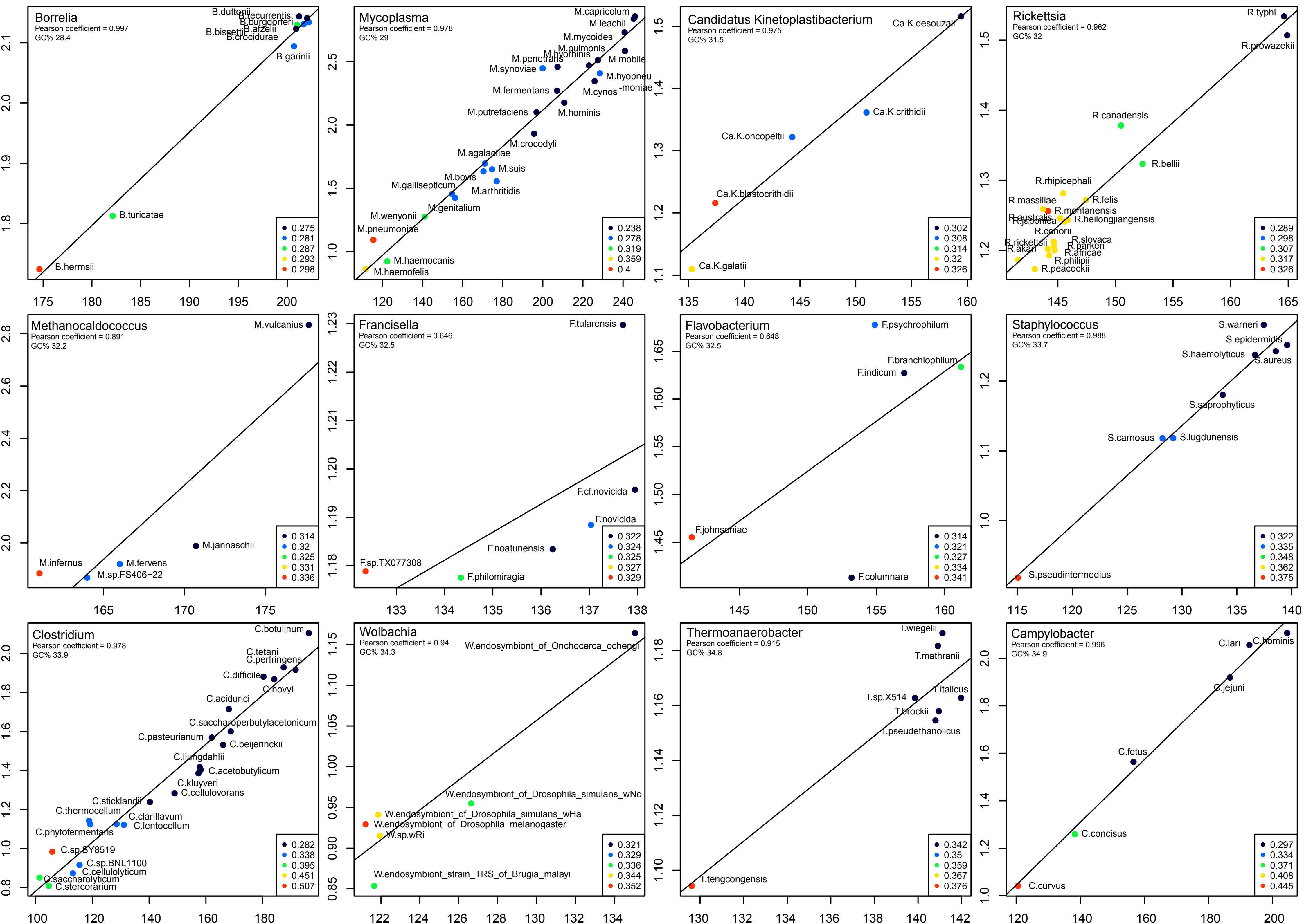
Borrelia
Pearson coefficient = 0.997
GC% 28.4
B.duttonii
B.recurrentis
B.burgdorferi
B.afzelii
B.bissettii
B.crocidurae
B.garinii
B.turicatae
B.hermsii
0.275
0.281
0.287
0.293
0.298
Mycoplasma
Pearson coefficient = 0.978
GC% 29
M.capricolum
M.leachii
M.mycoides
M.pulmonis
M.hyorhinis
M.penetrans
M.mobile
M.synoviae
M.hyopneu-moniae
M.fermentans
M.cynos
M.putrefaciens
M.hominis
M.crocodyli
M.agalactiae
M.suis
M.bovis
M.arthritidis
M.gallisepticum
M.genitalium
M.wenyonii
M.pneumoniae
M.haemocanis
M.haemofelis
0.238
0.278
0.319
0.359
0.4
Candidatus Kinetoplastibacterium
Pearson coefficient = 0.975
GC% 31.5
Ca.K.desouzaii
Ca.K.crithidii
Ca.K.oncopeltii
Ca.K.blastocrithidii
Ca.K.galatii
0.302
0.308
0.314
0.32
0.326
Rickettsia
Pearson coefficient = 0.962
GC% 32
R.typhi
R.prowazekii
R.canadensis
R.bellii
R.rhipicephali
R.massiliae
R.felis
R.montanensis
R.australis
R.japonica
R.heilongjiangensis
R.conorii
R.rickettsii
R.slovaca
R.parkeri
R.akari
R.africae
R.philipii
R.peacockii
0.289
0.298
0.307
0.317
0.326
Methanocaldococcus
Pearson coefficient = 0.891
GC% 32.2
M.vulcanius
M.jannaschii
M.infernus
M.fervens
M.sp.FS406−22
0.314
0.32
0.325
0.331
0.336
Francisella
Pearson coefficient = 0.646
GC% 32.5
F.tularensis
F.cf.novicida
F.novicida
F.noatunensis
F.sp.TX077308
F.philomiragia
0.322
0.324
0.325
0.327
0.329
Flavobacterium
Pearson coefficient = 0.648
GC% 32.5
F.psychrophilum
F.branchiophilum
F.indicum
F.johnsoniae
F.columnare
0.314
0.321
0.327
0.334
0.341
Staphylococcus
Pearson coefficient = 0.988
GC% 33.7
S.warneri
S.epidermidis
S.haemolyticus
S.aureus
S.saprophyticus
S.carnosus
S.lugdunensis
S.pseudintermedius
0.322
0.335
0.348
0.362
0.375
Clostridium
Pearson coefficient = 0.978
GC% 33.9
C.botulinum
C.tetani
C.perfringens
C.difficile
C.novyi
C.acidurici
C.saccharoperbutylacetonicum
C.pasteurianum
C.beijerinckii
C.ljungdahlii
C.acetobutylicum
C.kluyveri
C.cellulovorans
C.sticklandii
C.thermocellum
C.clariflavum
C.lentocellum
C.phytofermentans
C.sp.SY8519
C.sp.BNL1100
C.cellulolyticum
C.saccharolyticum
C.stercorarium
0.282
0.338
0.395
0.451
0.507
Wolbachia
Pearson coefficient = 0.94
GC% 34.3
W.endosymbiont_of_Onchocerca_ochengi
W.endosymbiont_of_Drosophila_simulans_wNo
W.endosymbiont_of_Drosophila_simulans_wHa
W.endosymbiont_of_Drosophila_melanogaster
W.sp.wRi
W.endosymbiont_strain_TRS_of_Brugia_malayi
0.321
0.329
0.336
0.344
0.352
Thermoanaerobacter
Pearson coefficient = 0.915
GC% 34.8
T.wiegelii
T.mathranii
T.sp.X514
T.italicus
T.brockii
T.pseudethanolicus
T.tengcongensis
0.342
0.35
0.359
0.367
0.376
Campylobacter
Pearson coefficient = 0.996
GC% 34.9
C.lari
C.hominis
C.jejuni
C.fetus
C.concisus
C.curvus
0.297
0.334
0.371
0.408
0.445

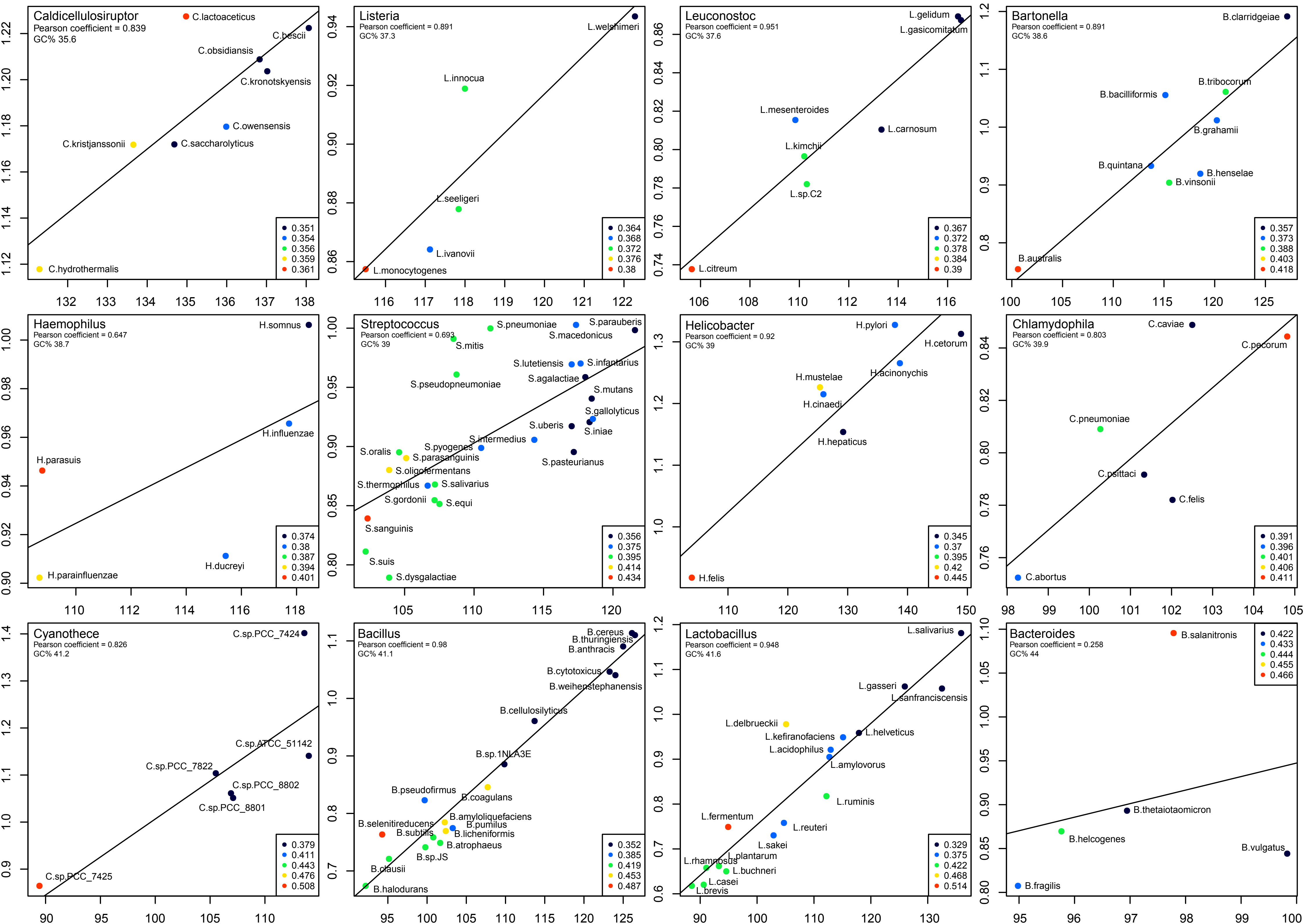

Caldicellulosiruptor
Pearson coefficient = 0.839
GC% 35.6
C.lactoaceticus
C.bescii
C.obsidiansis
C.kronotskyensis
C.owensensis
C.kristjanssonii
C.saccharolyticus
C.hydrothermalis
0.351
0.354
0.356
0.359
0.361
Listeria
Pearson coefficient = 0.891
GC% 37.3
L.welshimeri
L.innocua
L.seeligeri
L.ivanovii
L.monocytogenes
0.364
0.368
0.372
0.376
0.38
Leuconostoc
Pearson coefficient = 0.951
GC% 37.6
L.gelidum
L.gasicomitatum
L.mesenteroides
L.carnosum
L.kimchii
L.sp.C2
L.citreum
0.367
0.372
0.378
0.384
0.39
Bartonella
Pearson coefficient = 0.891
GC% 38.6
B.clarridgeiae
B.tribocorum
B.bacilliformis
B.grahamii
B.quintana
B.henselae
B.vinsonii
B.australis
0.357
0.373
0.388
0.403
0.418
Haemophilus
Pearson coefficient = 0.647
GC% 38.7
H.somnus
H.influenzae
H.parasuis
H.ducreyi
H.parainfluenzae
0.374
0.38
0.387
0.394
0.401
Streptococcus
Pearson coefficient = 0.693
GC% 39
S.pneumoniae
S.parauberis
S.macedonicus
S.mitis
S.lutetiensis
S.infantarius
S.agalactiae
S.pseudopneumoniae
S.mutans
S.gallolyticus
S.uberis
S.iniae
S.intermedius
S.pyogenes
S.oralis
S.parasanguinis
S.pasteurianus
S.oligofermentans
S.thermophilus
S.salivarius
S.gordonii
S.equi
S.sanguinis
S.suis
S.dysgalactiae
0.356
0.375
0.395
0.414
0.434
Helicobacter
Pearson coefficient = 0.92
GC% 39
H.pylori
H.cetorum
H.acinonychis
H.mustelae
H.cinaedi
H.hepaticus
H.felis
0.345
0.37
0.395
0.42
0.445
Chlamydophila
Pearson coefficient = 0.803
GC% 39.9
C.caviae
C.pecorum
C.pneumoniae
C.psittaci
C.felis
C.abortus
0.391
0.396
0.401
0.406
0.411
Cyanothece
Pearson coefficient = 0.826
GC% 41.2
C.sp.PCC_7424
C.sp.ATCC_51142
C.sp.PCC_7822
C.sp.PCC_8802
C.sp.PCC_8801
C.sp.PCC_7425
0.379
0.411
0.443
0.476
0.508
Bacillus
Pearson coefficient = 0.98
GC% 41.1
B.cereus
B.thuringiensis
B.anthracis
B.cytotoxicus
B.weihenstephanensis
B.cellulosilyticus
B.sp.1NLA3E
B.pseudofirmus
B.coagulans
B.selenitireducens
B.amyloliquefaciens
B.pumilus
B.subtilis
B.licheniformis
B.atrophaeus
B.sp.JS
B.clausii
B.halodurans
0.352
0.385
0.419
0.453
0.487
Lactobacillus
Pearson coefficient = 0.948
GC% 41.6
L.salivarius
L.gasseri
L.sanfranciscensis
L.delbrueckii
L.helveticus
L.kefiranofaciens
L.acidophilus
L.amylovorus
L.ruminis
L.fermentum
L.reuteri
L.sakei
L.rhamnosus
L.plantarum
L.buchneri
L.casei
L.brevis
0.329
0.375
0.422
0.468
0.514
Bacteroides
Pearson coefficient = 0.258
GC% 44
B.salanitronis
B.thetaiotaomicron
B.helcogenes
B.vulgatus
B.fragilis
0.422
0.433
0.444
0.455
0.466

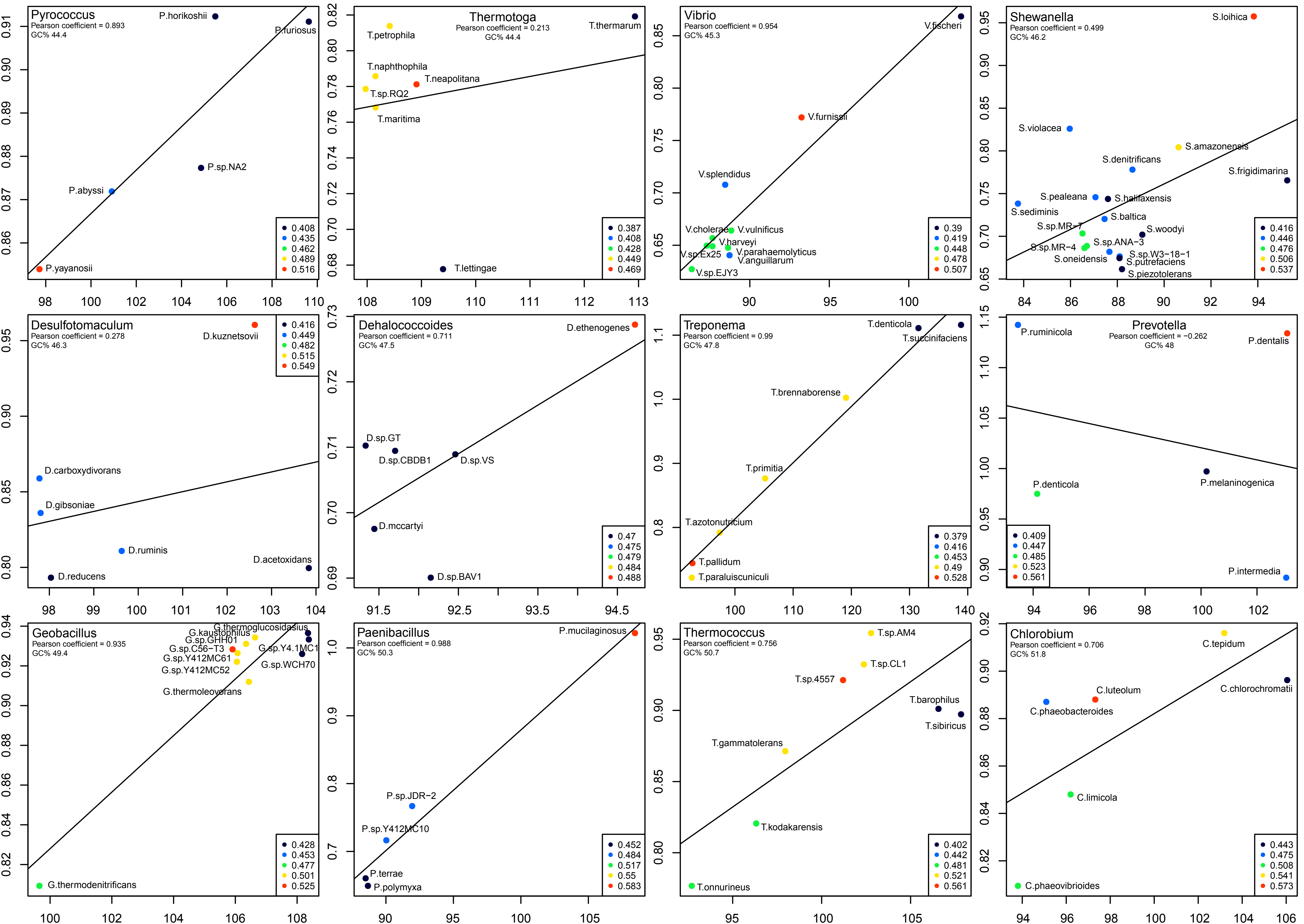
Pyrococcus
Pearson coefficient = 0.893
GC% 44.4
P.horikoshii
P.furiosus
P.sp.NA2
P.abyssi
P.yayanosii
Thermotoga
Pearson coefficient = 0.213
GC% 44.4
T.petrophila
T.thermarum
T.naphthophila
T.neapolitana
T.sp.RQ2
T.maritima
T.lettingae
Vibrio
Pearson coefficient = 0.954
GC% 45.3
V.fischeri
V.furnissii
V.splendidus
V.cholerae
V.vulnificus
V.harveyi
V.sp.Ex25
V.parahaemolyticus
V.anguillarum
V.sp.EJY3
Shewanella
Pearson coefficient = 0.499
GC% 46.2
S.loihica
S.violacea
S.amazonensis
S.denitrificans
S.frigidimarina
S.pealeana
S.halifaxensis
S.sediminis
S.baltica
S.sp.MR-7
S.woodyi
S.sp.ANA-3
S.sp.MR-4
S.sp.W3-18-1
S.oneidensis
S.putrefaciens
S.piezotolerans
Desulfotomaculum
Pearson coefficient = 0.278
GC% 46.3
D.kuznetsovii
D.carboxydivorans
D.gibsoniae
D.ruminis
D.acetoxidans
D.reducens
Dehalococcoides
Pearson coefficient = 0.711
GC% 47.5
D.ethenogenes
D.sp.GT
D.sp.CBDB1
D.sp.VS
D.mccartyi
D.sp.BAV1
Treponema
Pearson coefficient = 0.99
GC% 47.8
T.denticola
T.succinifaciens
T.brennaborense
T.primitia
T.azotonutricium
T.pallidum
T.paraluiscuniculi
Prevotella
Pearson coefficient = −0.262
GC% 48
P.ruminicola
P.dentalis
P.melaninogenica
P.denticola
P.intermedia
Geobacillus
Pearson coefficient = 0.935
GC% 49.4
G.thermoglucosidasius
G.kaustophilus
G.sp.GHH01
G.sp.C56−T3
G.sp.Y4.1MC1
G.sp.Y412MC61
G.sp.WCH70
G.sp.Y412MC52
G.thermoleovorans
G.thermodenitrificans
Paenibacillus
Pearson coefficient = 0.988
GC% 50.3
P.mucilaginosus
P.sp.JDR−2
P.sp.Y412MC10
P.terrae
P.polymyxa
Thermococcus
Pearson coefficient = 0.756
GC% 50.7
T.sp.AM4
T.sp.CL1
T.sp.4557
T.barophilus
T.sibiricus
T.gammatolerans
T.kodakarensis
T.onnurineus
Chlorobium
Pearson coefficient = 0.706
GC% 51.8
C.tepidum
C.luteolum
C.chlorochromatii
C.phaeobacteroides
C.limicola
C.phaeovibrioides

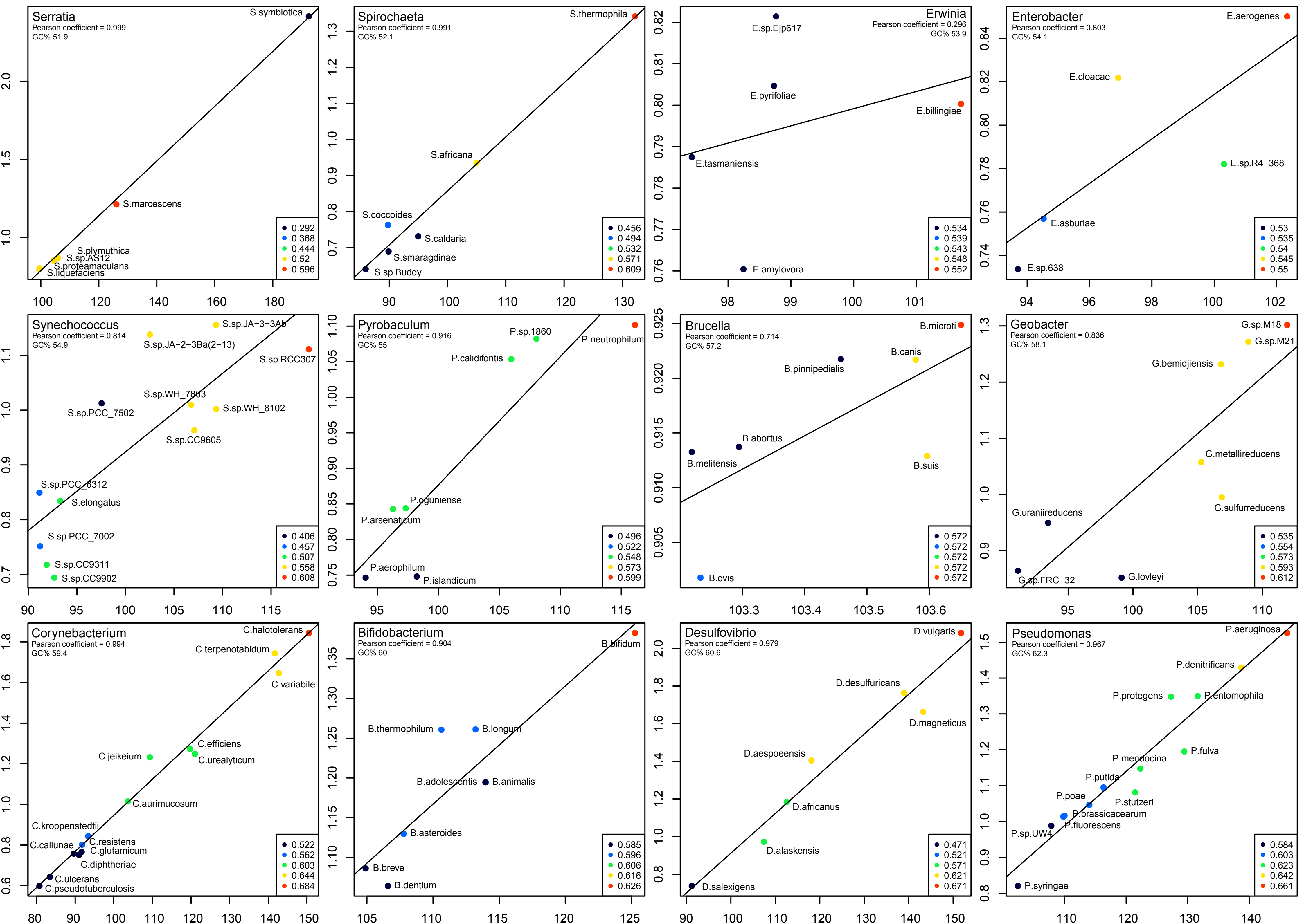

Serratia
Pearson coefficient = 0.999
GC% 51.9
Spirochaeta
Pearson coefficient = 0.991
GC% 52.1
Erwinia
Pearson coefficient = 0.296
GC% 53.9
Enterobacter
Pearson coefficient = 0.803
GC% 54.1
Synechococcus
Pearson coefficient = 0.814
GC% 54.9
Pyrobaculum
Pearson coefficient = 0.916
GC% 55
Brucella
Pearson coefficient = 0.714
GC% 57.2
Geobacter
Pearson coefficient = 0.836
GC% 58.1
Corynebacterium
Pearson coefficient = 0.994
GC% 59.4
Bifidobacterium
Pearson coefficient = 0.904
GC% 60
Desulfovibrio
Pearson coefficient = 0.979
GC% 60.6
Pseudomonas
Pearson coefficient = 0.967
GC% 62.3

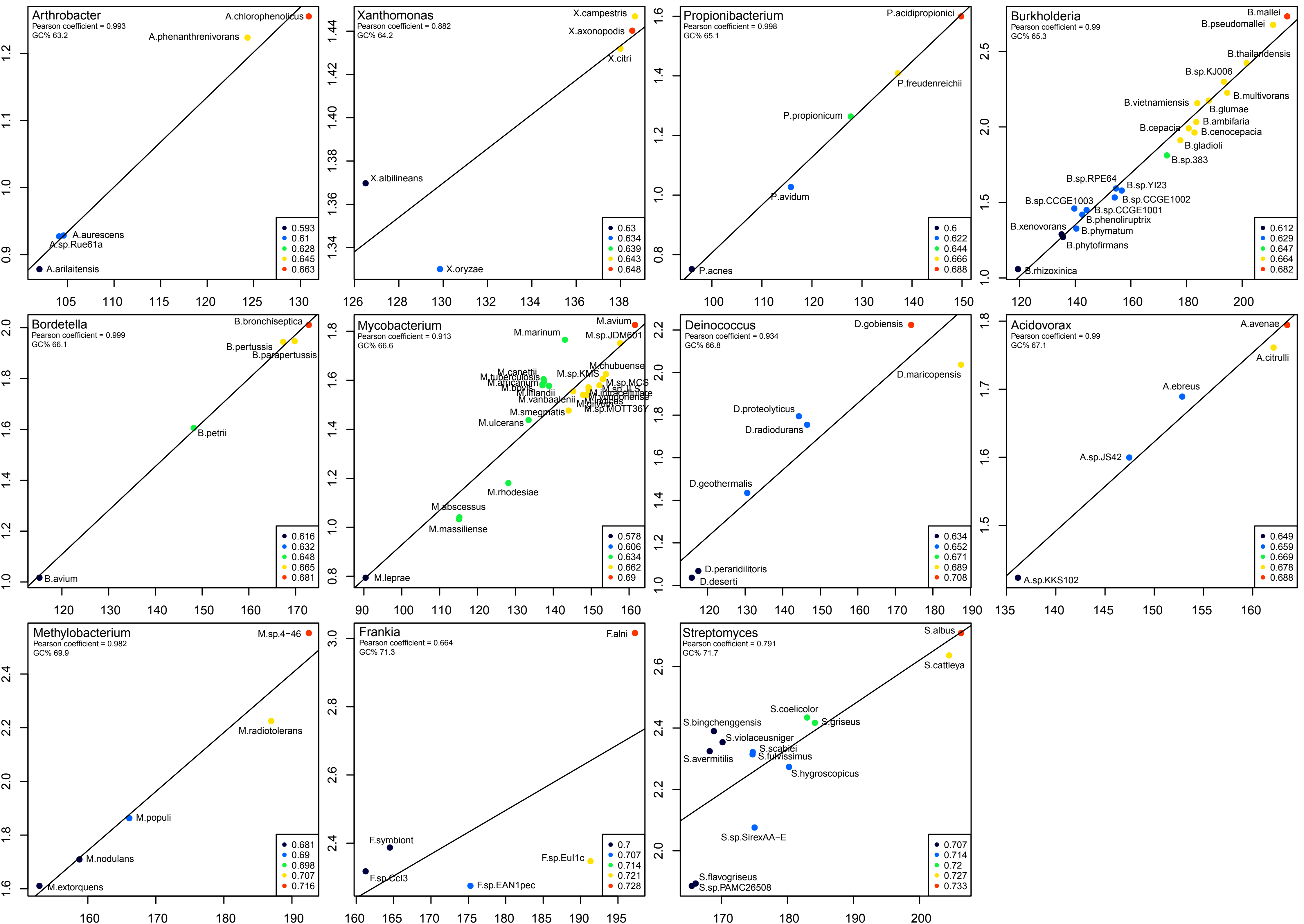
Arthrobacter
Pearson coefficient = 0.993
GC% 63.2
A.chlorophenolicus
A.phenanthrenivorans
A.aurescens
A.sp.Rue61a
A.arilaitensis
0.593 0.61 0.628 0.645 0.663
Xanthomonas
Pearson coefficient = 0.882
GC% 64.2
X.campestris
X.axonopodis
X.citri
X.albilineans
X.oryzae
0.63 0.634 0.639 0.643 0.648
Propionibacterium
Pearson coefficient = 0.998
GC% 65.1
P.acidipropionici
P.freudenreichii
P.propionicum
P.avidum
P.acnes
0.6 0.622 0.644 0.666 0.688
Burkholderia
Pearson coefficient = 0.99
GC% 65.3
B.mallei
B.pseudomallei
B.thailandensis
B.sp.KJ006
B.multivorans
B.vietnamiensis
B.glumae
B.ambifaria
B.cepacia
B.cenocepacia
B.gladioli
B.sp.383
B.sp.RPE64
B.sp.YI23
B.sp.CCGE1003
B.sp.CCGE1002
B.sp.CCGE1001
B.phenoliruptrix
B.xenovorans
B.phymatum
B.phytofirmans
B.rhizoxinica
0.612 0.629 0.647 0.664 0.682
Bordetella
Pearson coefficient = 0.999
GC% 66.1
B.bronchiseptica
B.pertussis
B.parapertussis
B.petrii
B.avium
0.616 0.632 0.648 0.665 0.681
Mycobacterium
Pearson coefficient = 0.913
GC% 66.6
M.avium
M.marinum
M.sp.JDM601
M.chubuense
M.canettii
M.tuberculosis
M.sp.KMS
M.africanum
M.sp.MCS
M.bovis
M.sp.JLS
M.liflandii
M.intracellulare
M.vanbaalenii
M.yongonense
M.indicus
M.gilvum
M.sp.MOTT36Y
M.smegmatis
M.ulcerans
M.rhodesiae
M.abscessus
M.massiliense
M.leprae
0.578 0.606 0.634 0.662 0.69
Deinococcus
Pearson coefficient = 0.934
GC% 66.8
D.gobiensis
D.maricopensis
D.proteolyticus
D.radiodurans
D.geothermalis
D.peraridilitoris
D.deserti
0.634 0.652 0.671 0.689 0.708
Acidovorax
Pearson coefficient = 0.99
GC% 67.1
A.avenae
A.citrulli
A.ebreus
A.sp.JS42
A.sp.KKS102
0.649 0.659 0.669 0.678 0.688
Methylobacterium
Pearson coefficient = 0.982
GC% 69.9
M.sp.4−46
M.radiotolerans
M.populi
M.nodulans
M.extorquens
0.681 0.69 0.698 0.707 0.716
Frankia
Pearson coefficient = 0.664
GC% 71.3
F.alni
F.symbiont
F.sp.EuI1c
F.sp.CcI3
F.sp.EAN1pec
0.7 0.707 0.714 0.721 0.728
Streptomyces
Pearson coefficient = 0.791
GC% 71.7
S.albus
S.cattleya
S.coelicolor
S.griseus
S.bingchenggensis
S.violaceusniger
S.scabiei
S.fulvissimus
S.avermitilis
S.hygroscopicus
S.sp.SirexAA−E
S.flavogriseus
S.sp.PAMC26508
0.707 0.714 0.72 0.727 0.733
